# Morphological Traits, Accumulation and Compartmentalization of Phenolic Compounds and Cadmium (CD^2+^) in *Linum usitatissimum* L. Seedlings Under In Vitro Conditions: A Comparative Study of Two Varieties

**DOI:** 10.3390/molecules31173064

**Published:** 2026-08-31

**Authors:** Evgenia A. Goncharuk, Tatyana L. Nechaeva, Maria Y. Zubova, Lyudmila V. Nazarenko, Natalia V. Zagoskina

**Affiliations:** 1 K.A. Timiryazev Institute of Plant Physiology, Russian Academy of Sciences, 127276 Moscow, Russia; nechaevatatyana.07@yandex.ru (T.L.N.); mariia.zubova@yandex.ru (M.Y.Z.); nzagoskina@mail.ru (N.V.Z.); 2 Department of Natural Sciences, Institute of Natural Sciences and Sports Technologies, Moscow City Teachers Training University, 105005 Moscow, Russia; nlv.mgpu@mail.ru

**Keywords:** *Linum usitatissimum* L., seedlings in vitro, phenolic compounds, heavy metals

## Abstract

Flax (*Linum usitatissimum* L.) is a multipurpose crop with considerable potential as a biotechnological platform for producing in vitro seedlings that serve both as sources of pharmacologically valuable phenolic compounds (PCs) and as potential biosorbents/chelators of heavy metals (HMs), including cadmium (Cd). The use of an in vitro culture system under strictly controlled conditions enables the identification of variety-specific responses in flax. The aim of this study was to compare the morphophysiological characteristics, the level of lipid peroxidation (LPO), and phenylpropanoid (PP) and flavonoid (FL) contents in in vitro seedlings of two flax varieties—fiber flax (FF) and oilseed flax (OF)—grown under control conditions and in the presence of Cd (60 and 100 μM). The stem mesostructure was also examined, as the stem is the principal organ responsible for metal transport via its vascular tissues. Our results showed that Cd exposure induced only minor changes in the morphophysiological characteristics of both flax varieties. Using a standardized in vitro system, we demonstrate for the first time that OF accumulated approximately 40% more Cd than FF at both metal concentrations. LPO, assessed by malondialdehyde (MDA) content, remained relatively stable across all treatments, except for a significant increase in OF exposed to 100 μM Cd. Analysis of the phenolic profile revealed that, in FF, the total phenolic content (TPC) and PP content increased relative to the control at 100 μM Cd, indicating the induction of PC-mediated antioxidant defense. In contrast, OF exhibited reduced PP and FL contents under both Cd treatments. Cytochemical analysis revealed a similar localization pattern for Cd and PC, with both accumulating predominantly in the stem epidermis of the in vitro-grown seedlings. However, the two flax types differed markedly in their accumulation profiles: OF accumulated higher levels of Cd, indicating its potential use as a biosorbent for this pollutant, whereas FF exhibited higher PC accumulation, suggesting its suitability as a natural source of PCs. These findings demonstrate that flax responses to Cd exposure are variety-specific.

## 1. Introduction

At present, one of the most effective approaches to studying plant metabolism and adaptation to stress factors is the use of in vitro culture systems as a biotechnological model, which offers numerous advantages over in vivo systems [1,2], including the ability to reduce pressure on natural resources; the ease of obtaining the required plant material for research regardless of environmental conditions or geographical and seasonal constraints; precise control of experimental conditions to optimize plant growth; and the accelerated production of various phytochemically active metabolites [1,2,3]. Particular emphasis should be placed on the value of in vitro cultures for accelerating the screening of economically important crops, including those with enhanced tolerance to anthropogenic stressors [4,5].

One of the major challenges of the modern era is the increasing anthropogenic pressure on the biosphere resulting from the rapid development of industry, transportation, and mineral extraction activities [6,7]. This has led to elevated concentrations of environmental pollutants, including heavy metals (HMs) [8]. Among them is cadmium (Cd), which can accumulate in plants as well as in the bodies of humans and animals, affecting numerous physiological processes [8,9,10]. In plants, Cd adversely affects water relations, photosynthesis, respiration, the transport of essential elements, the functioning of cellular membranes, enzyme activity, and other metabolic processes [10,11]. Human exposure to Cd from industrial and agricultural sources may cause cancer, affect the central nervous system, and lead to alterations in the functional activity of the liver, kidneys, mitochondria, and other cellular organelles [6,12].

Cd is known to act as a catalyst for the development of oxidative stress in plant cells through the excessive generation of reactive oxygen species (ROS), including the superoxide radical (O_2_•^−^), hydroxyl radical (HO•), hydrogen peroxide (H_2_O_2_), and singlet oxygen (^1^O_2_) [13,14]. The regulation of ROS levels is mediated by the antioxidant system, which comprises both high- and low-molecular-weight components [15,16]. Among the latter, phenolic compounds (PC)—one of the most widespread groups of plant secondary metabolites—play a critical role [17,18,19]. PC deactivate excited triplet states of molecules, act as free-radical scavengers, and act as chelating agents capable of binding transition-metal ions [16]. In recent years, plant-derived PC have found increasingly broad applications in medicine and the food industry owing to their antioxidant, anti-inflammatory, capillary-protective, antibacterial, and anticancer properties [20,21,22,23]. The effectiveness of protective functions is largely determined by the content and composition of these secondary metabolites, which vary depending on plant species and environmental conditions [24].

Flax (*Linum usitatissimum* L.) is among the unique versatile crops of considerable economic importance. It is widely used in the textile industry and animal feed production as a source of fiber and fiber-derived products, as well as in the paint and varnish industry for the production of industrial-grade oil. The pharmaceutical industry utilizes biologically valuable flaxseed oil enriched with α-linolenic acid and essential omega-3 and omega-6 fatty acids [25,26]. Of particular interest is the unique phytochemical profile of flax, which includes phenolic acids (ferulic, p-coumaric, and caffeic acids), flavonoids (orientin, vitexin, and isovitexin), and lignans (secoisolariciresinol diglucoside and its derivatives) [26,27,28,29]. Flax seeds and seedlings represent valuable raw materials for the pharmaceutical and nutraceutical industries due to their lignan content, as these compounds exhibit antimitotic, antiviral, and anticancer activities while also possessing the ability to adsorb HMs [26,29].

It should be noted that *L. usitatissimum* is represented by two principal botanical varieties—fiber flax (var. elongata) and oilseed flax (var. intermedia)—which differ in growth rate, developmental timing, biosynthetic processes, and photosynthetic productivity [30,31,32].

Despite the recognized importance of PC as biologically active plant metabolites involved in adaptation to a wide range of stress factors, including HMs, information on their accumulation in these two flax varieties, which differ in architecture and metabolic characteristics, remains highly fragmentary. The ability of flax to absorb heavy metals is well known, but the literature contains virtually no comparative studies on how different breeding approaches—oilseed (focusing on a high rate of dry matter accumulation) and textile (oriented towards producing high-quality fiber)—influence the choice of physiological and biochemical strategies for flax plant adaptation under toxic stress [25,26]. Furthermore, there is virtually no comparative data on the effects of moderate (60 μM) and high (100 μM) concentrations of cadmium on protective systems (which include, in addition to high-molecular-weight components, a pronounced low-molecular-weight defense pathway) in these varieties in isolated culture. This issue is particularly relevant because global flax cultivation often occurs in environmentally unfavorable areas—in soils contaminated with HMs, including Cd [33,34,35]. Previous studies have reported varietal differences in Cd accumulation and distribution among plant tissues [25,36], as well as Cd-induced changes in plant cell meso- and ultrastructure [37,38,39]. According to the literature, the role of the ABC and HMA gene families as key regulators of Cd accumulation is well known [40], as is the relationship between the expression of WOX genes and the activation of the phenylpropanoid pathway and cell wall formation in flax plants under stress conditions [41,42]. At the same time, current data on the effect of Cd on the metabolism of phenolic compounds in various representatives of the genus Linum remain scarce and mainly concern oilseed flax seeds [43]. This limits the understanding of the fundamental mechanisms of metal detoxification in the two varieties of this crop. Within the framework of this study, the focus is on the changes in the morphophysiological and biochemical parameters of flax seedlings under the influence of a pollutant.

Based on these considerations, we hypothesized that in vitro-grown seedlings of the two flax varieties may differ in a number of physiological and biochemical characteristics, including the accumulation of PC, as well as in their ability to employ distinct adaptive strategies in response to Cd exposure. Analyzing these features can help develop a biotechnological platform for producing in vitro flax cultures as a source of phenolic antioxidants and potential HM chelators, which could be used for the maintenance of human health in populations exposed to Cd stress. Consequently, this research is of considerable importance from both fundamental and applied perspectives.

The aim of the present study was to investigate the morphophysiological characteristics and mesostructure of in vitro-grown fiber flax (FF) and oilseed flax (OF) seedlings cultivated under control conditions and in the presence of Cd; to analyze metal and PC accumulation and compartmentalization in aboveground organs; and to assess the level of LPO. This integrated approach was designed to identify the specific responses of the two *L. usitatissimum* varieties to Cd exposure, thereby contributing to the evaluation of flax as a producer of biologically active metabolites as well as a potential biosorbent of HMs. The study aimed to identify species-specific adaptive fiber and oil flax varieties (*Linum usitatissimum* L.) at early stages of ontogenesis in vitro in response to moderate (60 μM) and supra-threshold (100 μM) exposure to cadmium.

## 2. Results

### 2.1. Morphophysiological Characteristics of In Vitro-Grown Flax Seedlings

**Figure 1:**
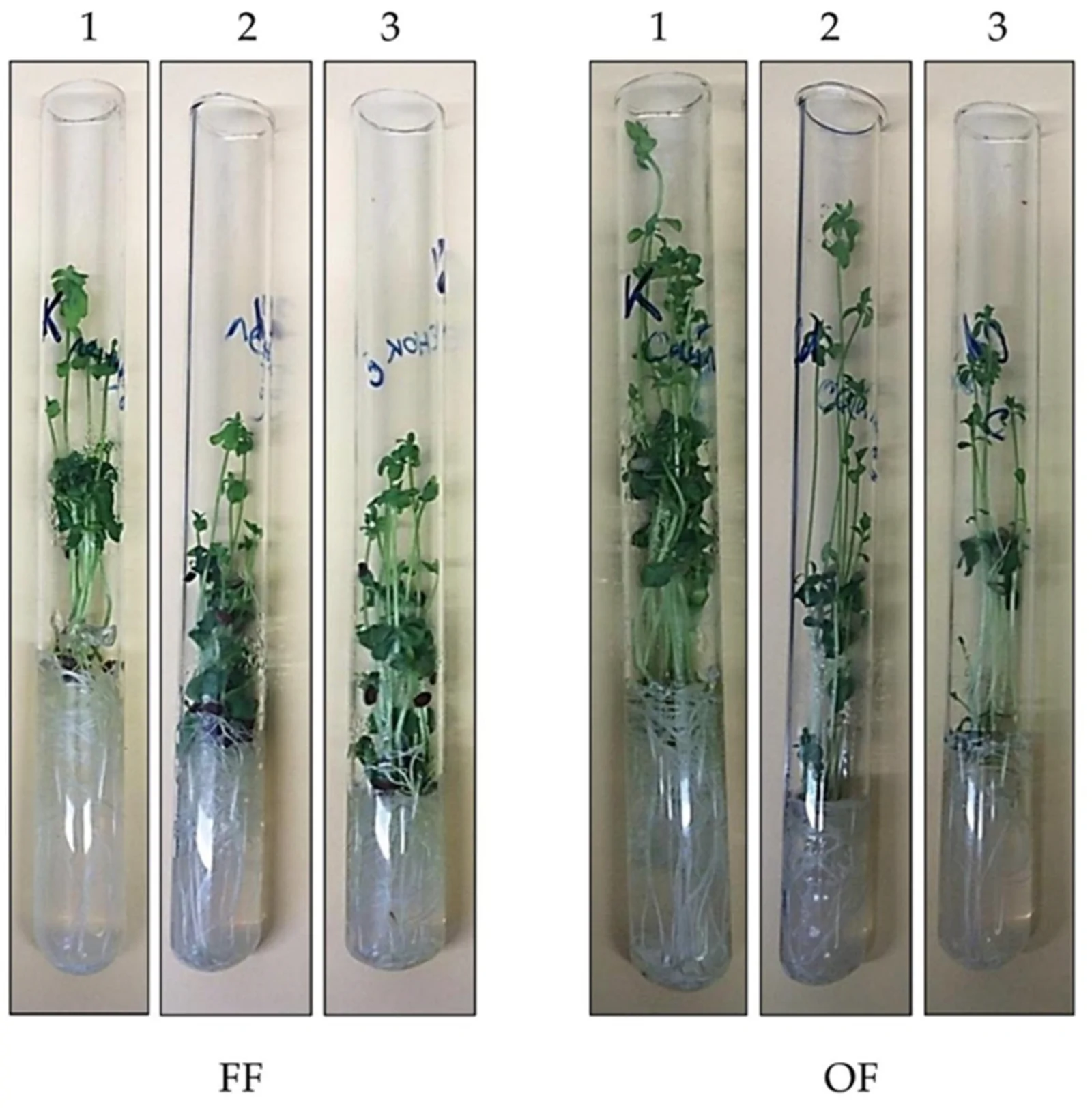
Appearance of in vitro fiber flax (FF) and oilseed flax (OF) seedlings cultivated under control conditions (1) or exposed to 60 μM Cd (2) and 100 μM Cd (3). Plant age: 21 days.

Morphophysiological parameters are important indicators of plant viability and responses to various environmental factors, including HMs [11]. In vitro-grown seedlings of the two flax varieties differed in their morphophysiological characteristics both when cultivated on the basal nutrient medium (control) and on media supplemented with different concentrations of Cd (treatments) (Figure 1, Table 1). In all experimental variants, FF exhibited lower growth parameters compared to OF.

In vitro-grown FF and OF seedlings cultivated under control conditions differed substantially in primary root length and shoot height (by 50%) and in the number of leaves (by 33%) (Table 1).

The presence of Cd in the nutrient media induced concentration-dependent changes in the morphometric parameters of the in vitro flax seedlings. At 60 μM Cd, the primary root length, shoot height, and leaf number in FF decreased by 12%, 10%, and 14%, respectively, relative to the control, whereas the corresponding reductions in OF were 13%, 17%, and 23%. At 100 μM Cd, these effects became more pronounced. In FF, the primary root length, shoot height, and leaf number were 37%, 30%, and 22% lower than the control values, respectively, while in OF the corresponding decreases were 35%, 30%, and 40%.

Determination of water content, an important indicator of the physiological status of plant tissues, revealed similar values in control seedlings of the two flax varieties (Table 1). Exposure to 60 and 100 μM Cd resulted in a decline in this parameter, amounting to 1.4% and 2.6% in FF and 3% and 4% in OF, respectively.

Among the variety-specific changes in morphometric characteristics observed under control conditions were primary root length, shoot height, and leafiness. All of these growth parameters were lower in FF than in OF. In the Cd-treated variants, these metrics exhibited concentration-dependent responses; however, their reduction relative to the control was more pronounced in OF than in FF, with the strongest effect observed at the higher Cd concentration. Tissue water content was similar in the two flax varieties and changed only slightly relative to the control in response to Cd exposure (Table 1).

It should be noted that no visible symptoms of chlorosis or tissue necrosis were observed in any of the experimental variants. This indicates that in vitro-grown seedlings of both flax varieties exhibited tolerance to Cd, despite its inhibitory and concentration-dependent effects on plant growth, which were more pronounced in OF.

### 2.2. Cadmium Content in In Vitro-Grown Flax Seedlings

**Figure 2:**
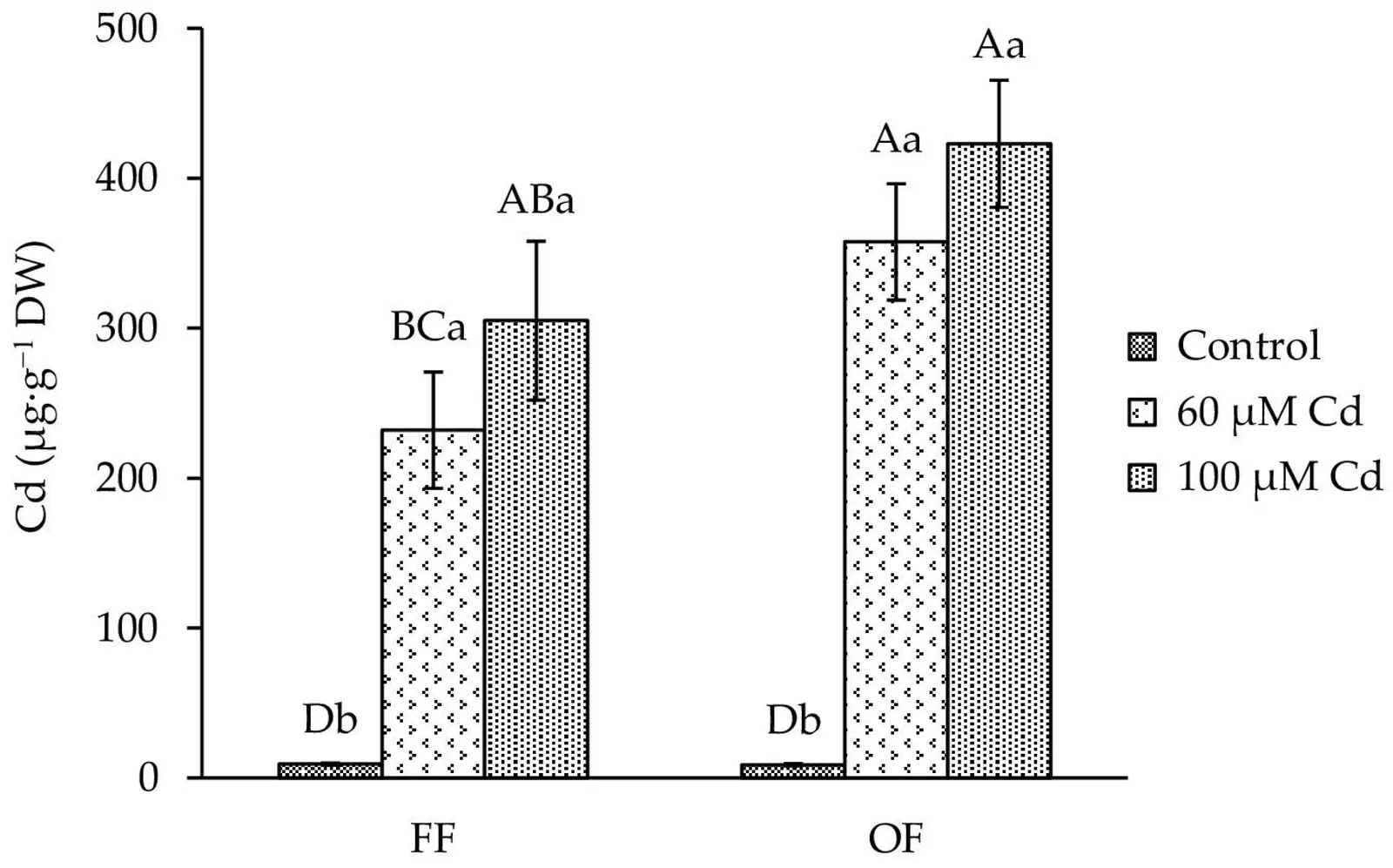
Cadmium (Cd) content in in vitro fiber flax (FF) and oilseed flax (OF) seedlings cultivated under control conditions (Control) or exposed to different Cd concentrations. Results are presented as mean ± standard deviation (SD), n = 3. Significant differences at *p* < 0.05 are indicated by different Latin letters: uppercase letters denote significant differences between flax varieties, arranged alphabetically from the highest to the lowest mean value, whereas lowercase letters denote significant differences among Cd treatments, arranged alphabetically from the highest to the lowest mean value.

The presence of Cd in the environment generally leads to its accumulation in plants, the extent of which depends on species-specific metabolic characteristics, as well as the concentration and duration of metal exposure [34]. Determination of Cd content in in vitro-grown seedlings of the two flax varieties revealed substantial differences in their capacity to accumulate the metal in aboveground organs (Figure 2). Cd accumulation in FF was statistically significantly lower than in OF, by 35% and 27% in the presence of 60 and 100 μM Cd in the nutrient medium, respectively.

Within each flax variety, Cd content did not differ significantly between the two Cd concentrations tested, indicating that the mechanisms responsible for metal uptake became saturated already at the lower concentration (60 μM).

### 2.3. Lipid Peroxidation in In Vitro-Grown Flax Seedlings

The content of malondialdehyde (MDA) is widely used as an indicator of LPO intensity, and its increase under stress conditions is considered a marker of oxidative stress [44,45].

**Figure 3:**
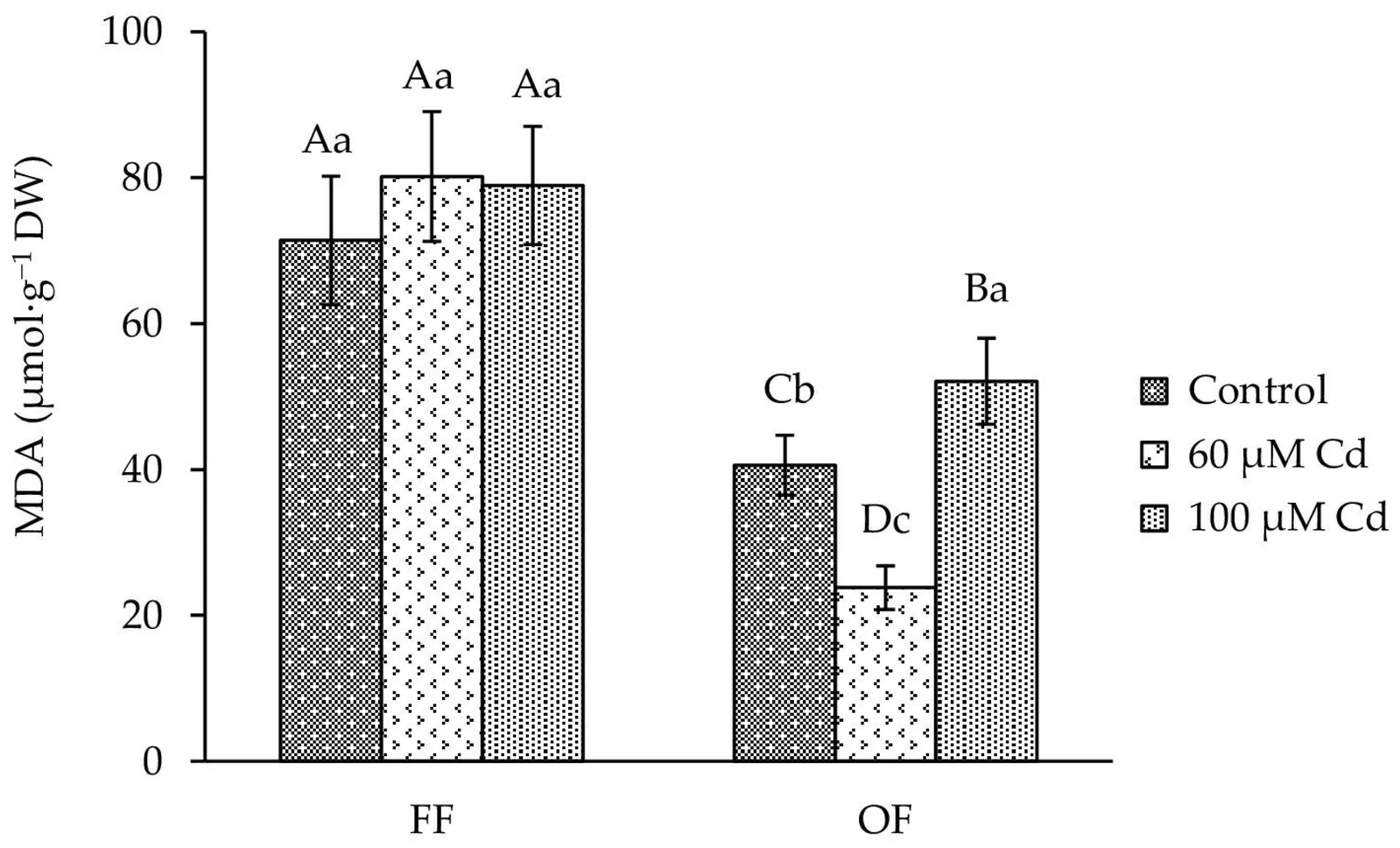
Malondialdehyde (MDA) content in in vitro fiber flax (FF) and oilseed flax (OF) seedlings cultivated under control conditions (Control) or exposed to different concentrations of cadmium (Cd). Results are presented as mean ± standard deviation (SD), n = 3. Significant differences at *p* < 0.05 are indicated by different Latin letters: uppercase letters denote significant differences between flax varieties, arranged alphabetically from the highest to the lowest mean value, whereas lowercase letters denote significant differences among Cd treatments, arranged alphabetically from the highest to the lowest mean value.

In the control seedlings, the MDA content in FF was approximately twofold higher than in OF (Figure 3), indicating differences in their basal LPO levels.

Exposure to Cd elicited distinct responses in the in vitro seedlings of the two flax varieties. In FF, no statistically significant changes in MDA content relative to the control were observed in the presence of the pollutant. A different pattern was registered in OF: exposure to 60 μM Cd resulted in a decrease in MDA content, whereas exposure to 100 μM Cd caused an increase. Relative to the control, MDA levels decreased by 41% at 60 μM Cd and increased by 28% at 100 μM Cd. These findings indicate that high metal concentrations trigger a stress response in OF seedlings, whereas no such response was detected in FF regardless of Cd concentration in the medium.

### 2.4. Phenolic Compound Accumulation Profile in In Vitro-Grown Flax Seedlings

PC are ubiquitous secondary metabolites with antioxidant activity, which play an important role in plant physiology, including adaptation to stressful environmental conditions [16,18].

**Figure 4:**
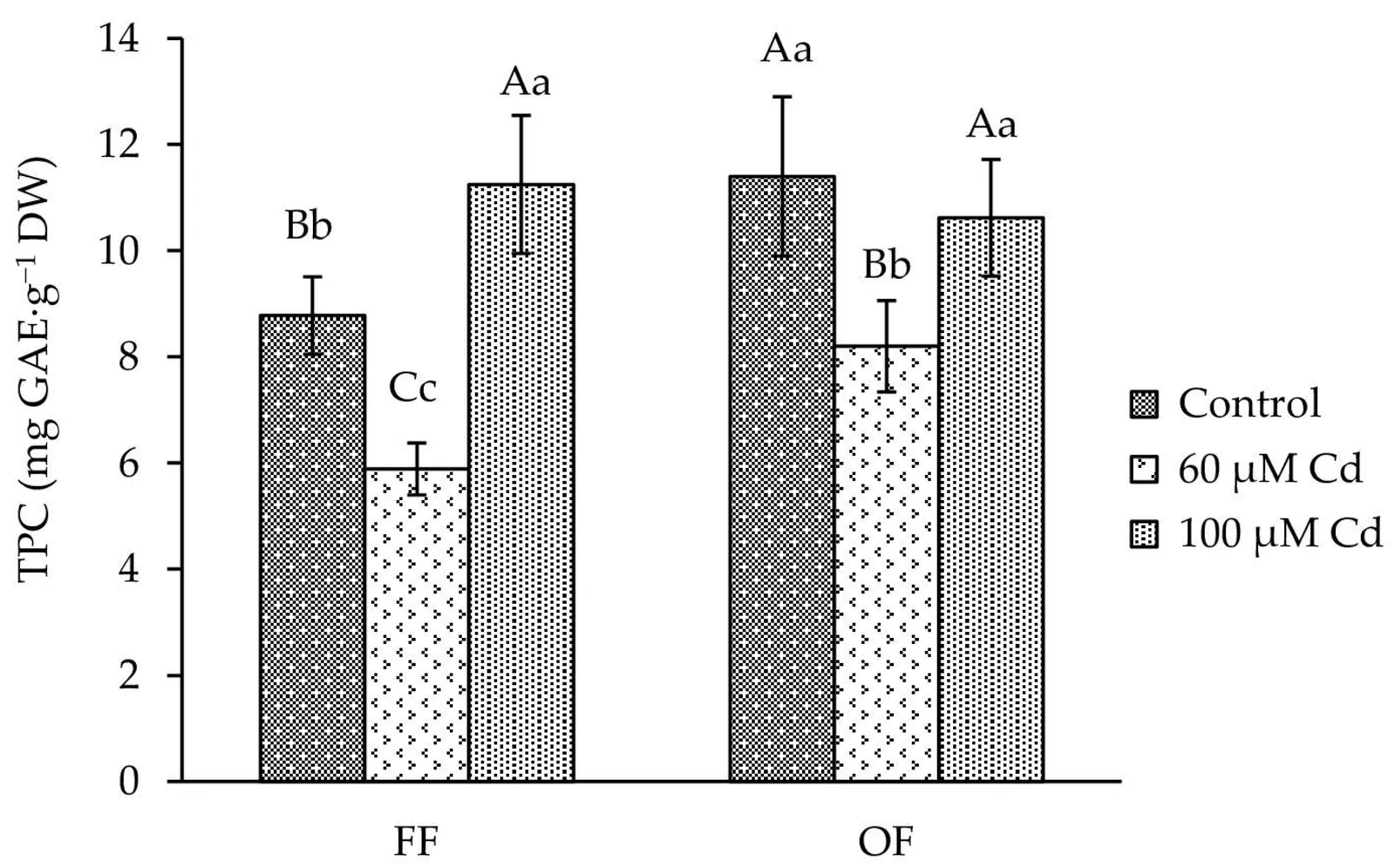
Total phenolic content (TPC) in in vitro fiber flax (FF) and oilseed flax (OF) seedlings cultivated under control conditions (Control) or exposed to different concentrations of cadmium (Cd). Results are presented as mean ± standard deviation (SD), n = 3. Significant differences at *p* < 0.05 are indicated by different Latin letters: uppercase letters denote significant differences between flax varieties, arranged alphabetically from the highest to the lowest mean value, whereas lowercase letters denote significant differences among Cd treatments, arranged alphabetically from the highest to the lowest mean value.

As an initial step, the total phenolic content (TPC) in the in vitro seedlings of the two flax varieties was analyzed to evaluate their capacity to accumulate these compounds (Figure 4).

Under control conditions, the TPC of in vitro-grown FF seedlings was 30% lower compared to OF. Exposure to Cd altered this parameter in both varieties. In FF, TPC decreased by 34% relative to the control at 60 μM Cd, whereas at 100 μM Cd it exceeded the control level by 28%. A similar trend was observed in OF at the lower metal concentration, with TPC decreasing by 28%; however, at the higher concentration, TPC remained comparable to the control level.

**Figure 5:**
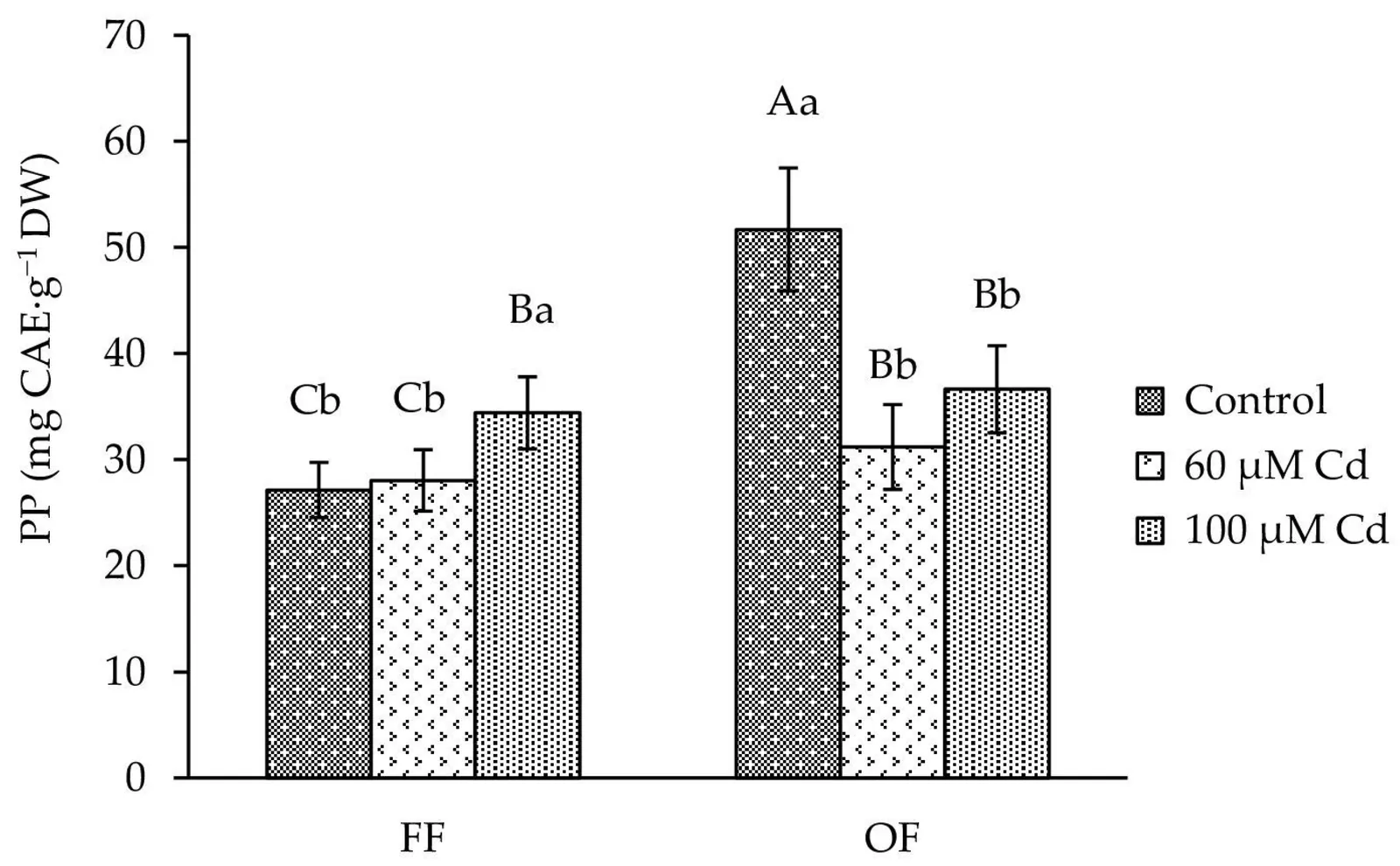
Phenylpropanoid content (PP) in in vitro fiber flax (FF) and oilseed flax (OF) seedlings cultivated under control conditions (Control) or exposed to different concentrations of cadmium (Cd). Results are presented as mean ± standard deviation (SD), n = 3. Significant differences at *p* < 0.05 are indicated by different Latin letters: uppercase letters denote significant differences between varieties, arranged alphabetically from the highest to the lowest mean value, whereas lowercase letters denote significant differences among Cd treatments, arranged alphabetically from the highest to the lowest mean value.

Phenylpropanoids (PPs), which belong to the group of biogenetically early phenolic metabolites, may either accumulate in plant cells or serve as precursors in flavonoid biosynthesis [16,46]. Analysis of their content revealed that, under control conditions, in vitro FF seedlings contained nearly twofold lower levels of these compounds than OF (Figure 5).

Exposure to Cd revealed distinct patterns of PP accumulation in the in vitro seedlings of the two flax varieties. In FF, no changes in PP content were observed at 60 μM Cd, whereas exposure to 100 μM Cd increased their level by 27%. A contrasting trend emerged in OF seedlings, with these metabolites reduced by 40% and 29% relative to the control following exposure to 60 and 100 μM Cd, respectively, reaching nearly identical levels under both treatments.

**Figure 6:**
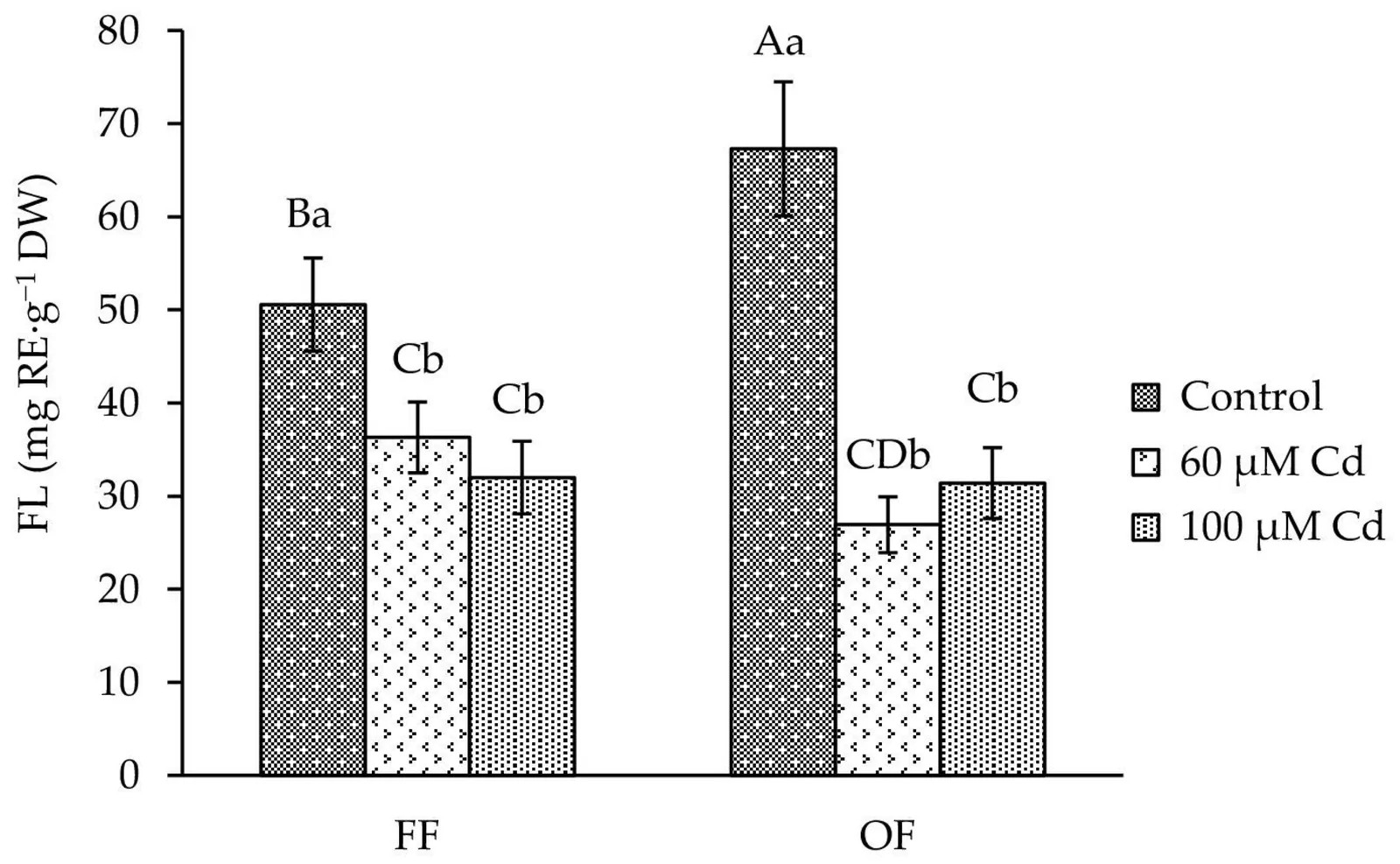
Flavonoid content (FL) in in vitro fiber flax (FF) and oilseed flax (OF) seedlings cultivated under control conditions (Control) or exposed to different concentrations of cadmium (Cd). Results are presented as mean ± standard deviation (SD), n = 3. Significant differences at *p* < 0.05 are indicated by different Latin letters: uppercase letters denote significant differences between varieties, arranged alphabetically from the highest to the lowest mean value, whereas lowercase letters denote significant differences among Cd treatments, arranged alphabetically from the highest to the lowest mean value.

Flavonoids (FLs) are among the most widespread PC found in plants [47]. In flax seedlings cultivated in vitro under control conditions, FL content in OF exceeded that in FF by 33% (Figure 6).

Cd exposure reduced the accumulation of these secondary metabolites in the in vitro-grown seedlings of both flax varieties. In FF, FL content was 28% and 35% lower than the control at 60 and 100 μM Cd, respectively. A similar but more pronounced trend was observed in OF, with this parameter decreasing by 60% and 53% at the two corresponding Cd concentrations. These changes resulted in almost identical FL levels in all Cd-treated variants of the in vitro seedlings.

**Figure 7:**
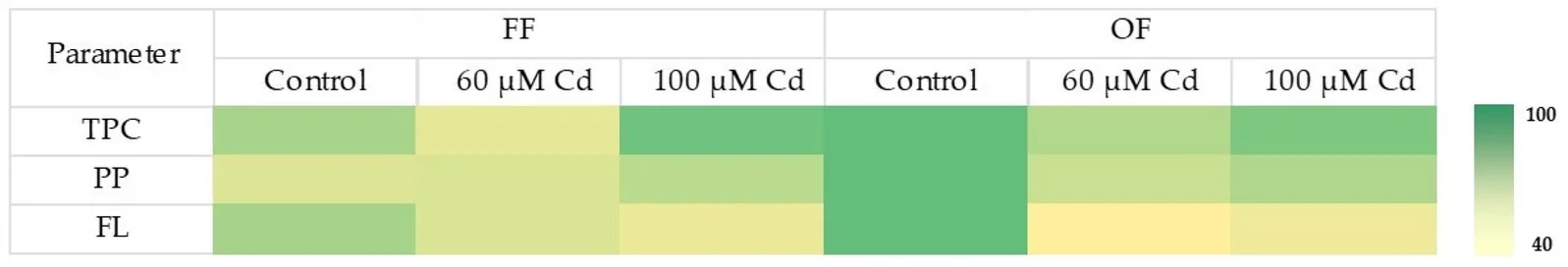
Heat map showing the accumulation of total phenolic compounds (TPC), phenylpropanoids (PPs), and flavonoids (FLs) in in vitro fiber flax (FF) and oilseed flax (OF) seedlings cultivated under control conditions (Control) or exposed to different concentrations of cadmium (Cd). Values in each row (parameter) were normalized to the maximum value, which was set at 100%. Color intensity indicates the relative level of each parameter across the different samples.

Visualization of the PC accumulation data in the form of a heat map highlights both variety-specific and concentration-dependent changes in the in vitro FF and OF seedlings cultivated under control conditions (Control) or exposed to different concentrations of cadmium (Cd) (Figure 7).

The variety-specific differences included a higher PP content in OF than in FF under control conditions, as well as a higher FL content in OF compared to FF in the control, with FL levels decreasing to a similar extent in both varieties upon Cd exposure. Concentration-dependent changes were observed for TPC and PP in FF, both of which increased relative to the control at 100 μM Cd. Overall, these results indicate, first, that the two flax varieties exhibit differential responses to Cd exposure with respect to the accumulation of different classes of phenolic metabolites. Second, they show that Cd exposure generally led to a reduction in the levels of these compounds, with the effect being more pronounced in OF.

### 2.5. Anatomical and Morphological Characteristics of In Vitro-Grown Flax Seedlings

An important aspect of evaluating plant tissues, including under the influence of external factors, is the analysis of their mesostructure using light microscopy techniques [48,49,50]. However, information on such studies in in vitro-grown seedlings of the genus Linum is currently lacking, which prompted the present investigation.

**Figure 8:**
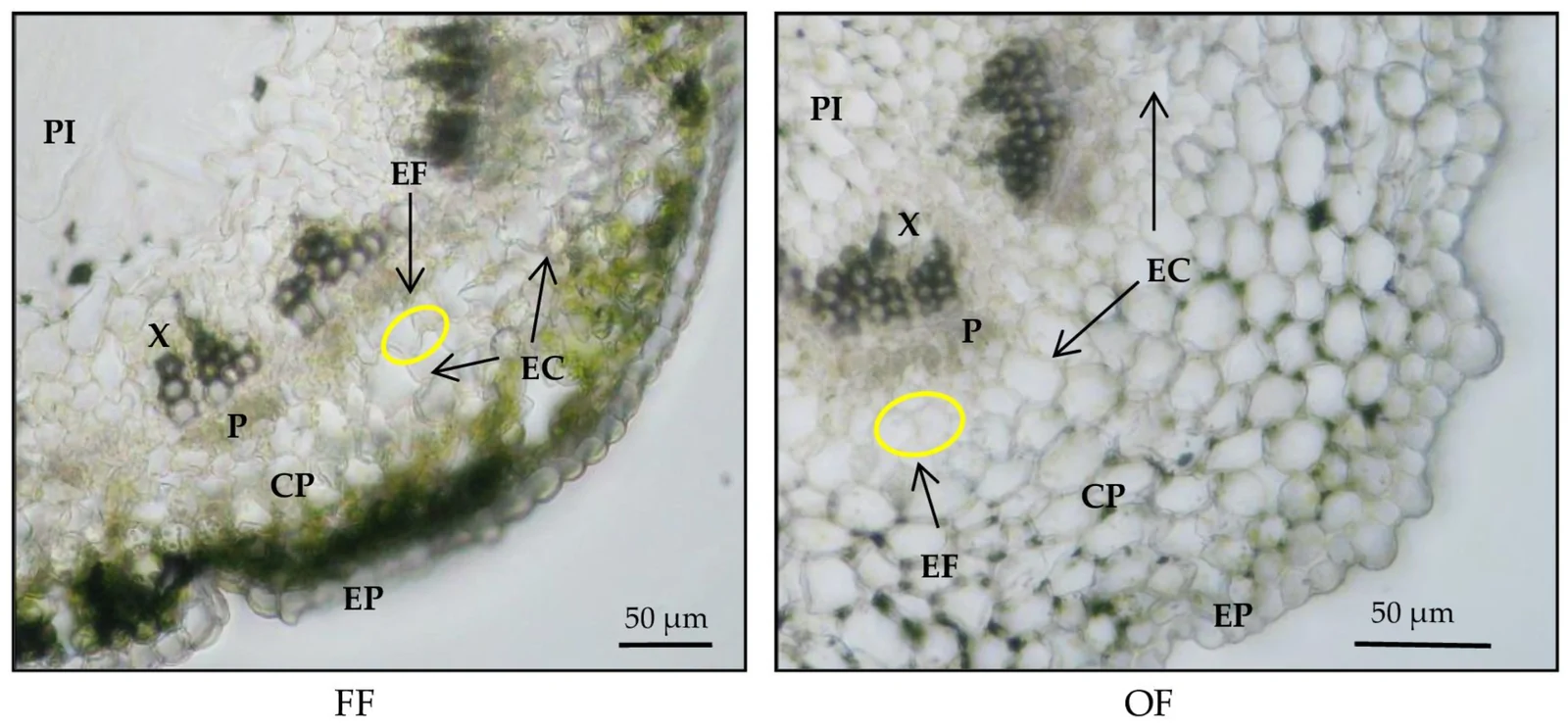
Cross sections of the stems of in vitro fiber flax (FF) and oilseed flax (OF) seedlings cultivated under control conditions. Abbreviations: epidermis (EP); cortical parenchyma (CP); endodermal cells (ECs); phloem (P); xylem (X); region of developing elementary fibers (EFs; highlighted in yellow); pith (PI).

Cross sections of the stems of in vitro seedlings revealed the following anatomical-topographical zones: epidermis (EP), cortical parenchyma (CP) with endodermal cells (ECs) and developing elementary fibers (EFs), phloem (P), xylem (X), and pith (PI) (Figure 8).

In the peripheral (outer) region of the stem, in vitro flax seedlings possessed a single-layered EP. In FF, epidermal cells were predominantly oval in shape, whereas in OF both oval and rounded cells were observed. Beneath the EP was the CP layer, composed of thin-walled cells of oval and tangentially elongated shape. In FF, this tissue consisted of 4–5 cell layers, whereas in OF it comprised 8–9 layers.

In both flax varieties, the P occupied a narrower region than the X and contained a small number of cells initiating EF formation. These developing fibers were located between the CP and X, opposite the xylem rays.

The next stage of the morphometric analysis involved determining the mean cell area of different stem tissues in the in vitro-grown seedlings (Table 2). To this end, tissue types of particular functional and developmental significance for plants of the genus Linum were examined, including the dermal tissue (EP), ground tissue (CP, including EC of the developing endodermis), and mechanical tissue (sclerenchyma), represented by developing EF that subsequently give rise to bast fiber bundles.

Under control conditions, the two flax varieties exhibited similar mean cell areas for each tissue type. The smallest cell areas were observed in the EP, and the largest in the EF, with the difference between these tissues approaching twofold. The presence of Cd did not result in significant changes in mean cell area, except in OF exposed to 100 μM Cd, where the mean area of EC and EF decreased by 28% and 35%, respectively, relative to the control.

**Figure 9:**
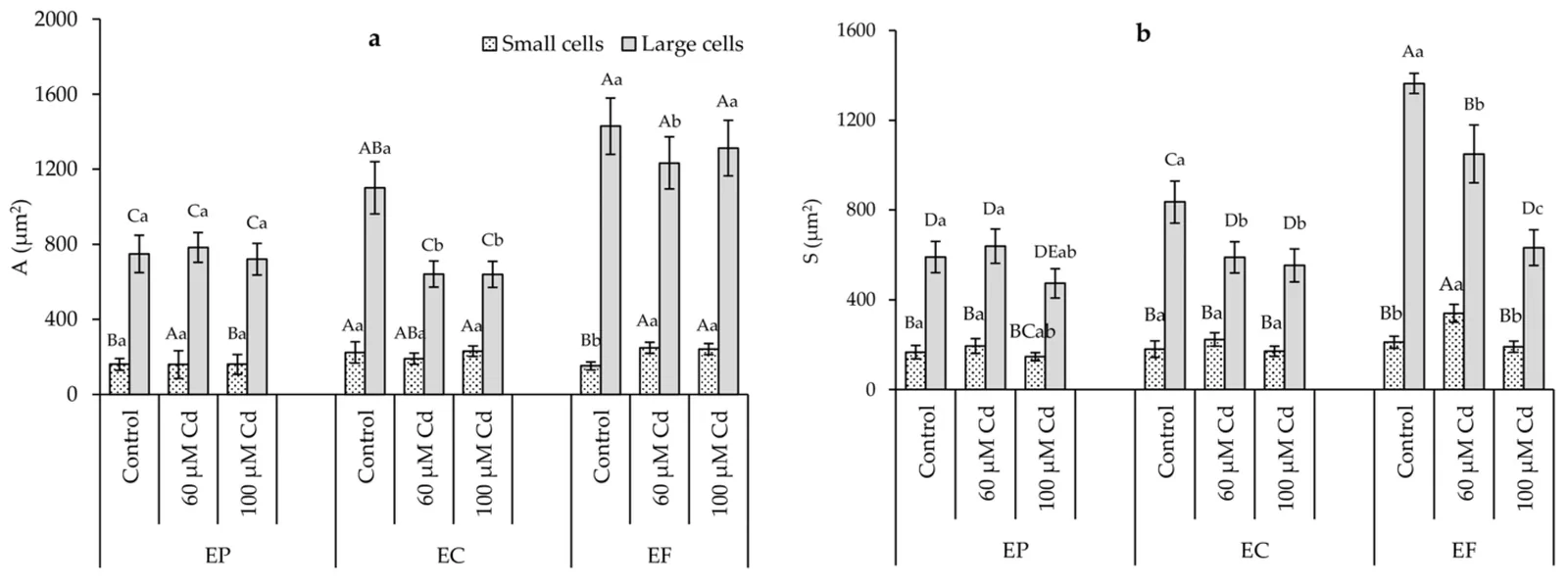
Area (A) of small and large cells in different stem tissues of in vitro fiber flax (FF) (**a**) and oilseed flax (OF) (**b**) seedlings cultivated under control conditions (Control) or exposed to different concentrations of cadmium (Cd). Results are presented as mean ± standard deviation (SD), n = 100. Significant differences at *p* < 0.05 are indicated by different Latin letters: uppercase letters denote significant differences between varieties, arranged alphabetically from the highest to the lowest mean value, whereas lowercase letters denote significant differences among cadmium treatments, arranged alphabetically from the highest to the lowest mean value. Abbreviations: epidermis (EP); endodermal cells (ECs); elementary fibers (EFs).

The tissues of young flax stems are characterized by the presence of both small and large cells, whose functional roles are associated with metabolic activity or the provision of mechanical support [51]. Morphometric analysis revealed differences in this parameter in the EP, EC, and EF of in vitro-grown seedlings of the two flax varieties cultivated under control conditions and exposed to Cd (Figure 9).

In flax seedlings grown under control conditions, differences were only observed in the area of large cells forming the various tissue types. These differences were particularly pronounced in the EP and EC, with cell area in FF exceeding that in OF by 15% and 20%, respectively (Figure 9a,b).

Exposure to Cd altered the area of stem tissue-forming cells in the in vitro seedlings of both flax varieties relative to their respective controls. In FF, reductions were only observed in the area of large EC (by 45% at both Cd concentrations) and large EF (by 12%, but only at 60 μM Cd). The area of both large and small EP remained unchanged (Figure 9a).

A different pattern was observed in OF, where a significant reduction in cell area was recorded for all tissue types relative to the control. The area of both large and small EC decreased by 17% and 20%, respectively, but only at 100 μM Cd. The area of large EC declined by 20% and 30% at the two Cd concentrations. The area of large EF decreased by 23% and 51% at 60 and 100 μM Cd, respectively, whereas the area of small EF increased at 60 μM Cd (Figure 9b).

**Figure 10:**
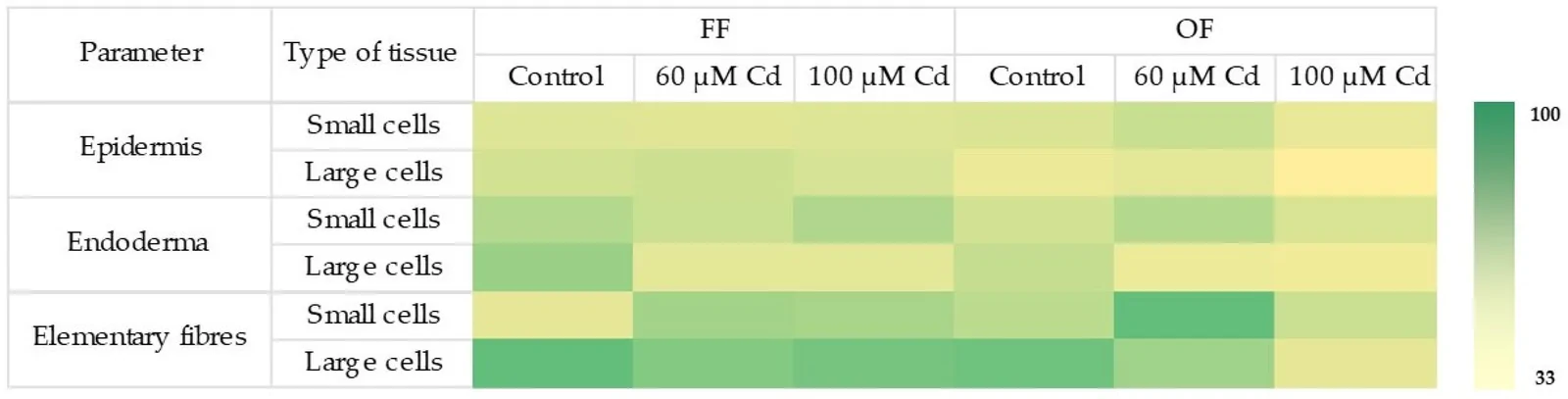
Heat map of changes in the area of large and small stem cells in in vitro fiber flax (FF) and oilseed flax (OF) seedlings cultivated under control conditions (Control) or exposed to different concentrations of cadmium (Cd). Values in each row (parameter) were normalized to the maximum value, which was set at 100% separately for large and small cells. Color intensity indicates the relative level of each parameter across the different samples.

As shown in Figure 10, under control conditions, the two flax varieties exhibited variety- and tissue-specific differences only in the area of large cells. These differences were most pronounced in the EP and EC of FF. Exposure to Cd induced changes in the analyzed parameters that were variety- and tissue-specific, and concentration-dependent. In FF, these effects were limited to reductions in the area of large EC and EF relative to the control. In OF, the area of all tissue types decreased. This reduction involved both large and small EP cells at the higher Cd concentration, but only large cells in EC and EF at both concentrations. At the same time, exposure to the lower Cd concentration resulted in an increase in the area of small EF.

All these findings demonstrate both similarities and differences in the anatomical and morphological characteristics of in vitro-grown seedlings of the two flax varieties at the mesostructural level, as well as concentration-dependent modifications induced by Cd exposure.

### 2.6. Cytochemical Characteristics of In Vitro-Grown Flax Seedlings

The application of cytochemical methods is an important approach for investigating various aspects of plant biology, including adaptation to HM stress [52]. Once taken up by plants, Cd is known to be distributed unevenly among different cellular compartments, and this process is strongly influenced by species-specific characteristics as well as the duration of stress exposure [53].

**Figure 11:**
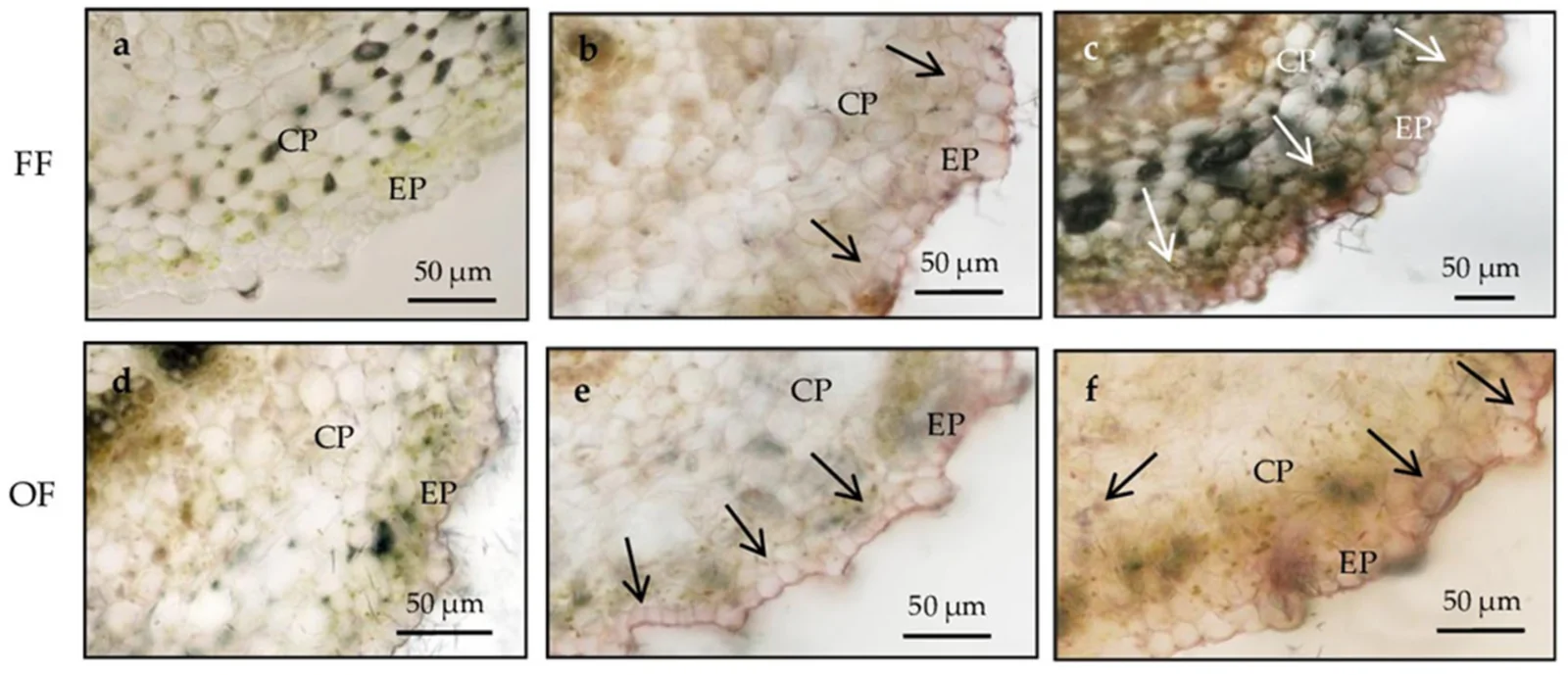
Localization of cadmium (Cd) in the stems of in vitro fiber flax (FF) (**a**–**c**) and oilseed flax (OF) (**d**–**f**) seedlings cultivated under control conditions (**a**,**d**) or exposed to 60 μM (**b**,**e**) and 100 μM (**c**,**f**) Cd. Sites of metal localization are indicated by arrows. Abbreviations: epidermis (EP); cortical parenchyma (CP).

In the stems of in vitro seedlings of both flax varieties, Cd localization, as detected by the histochemical dithizone reaction, was observed primarily in the cell walls of the EP and sporadically in CP cells, with staining intensity increasing at the higher Cd concentration (100 μM) (Figure 11).

A greater capacity for pollutant accumulation was observed in OF, which was consistent with the quantitative data on Cd content (Figure 2). These results confirm Cd uptake by in vitro-grown seedlings of both flax varieties and its localization predominantly in the outer stem tissues, with more pronounced effects at the higher Cd concentration.

**Figure 12:**
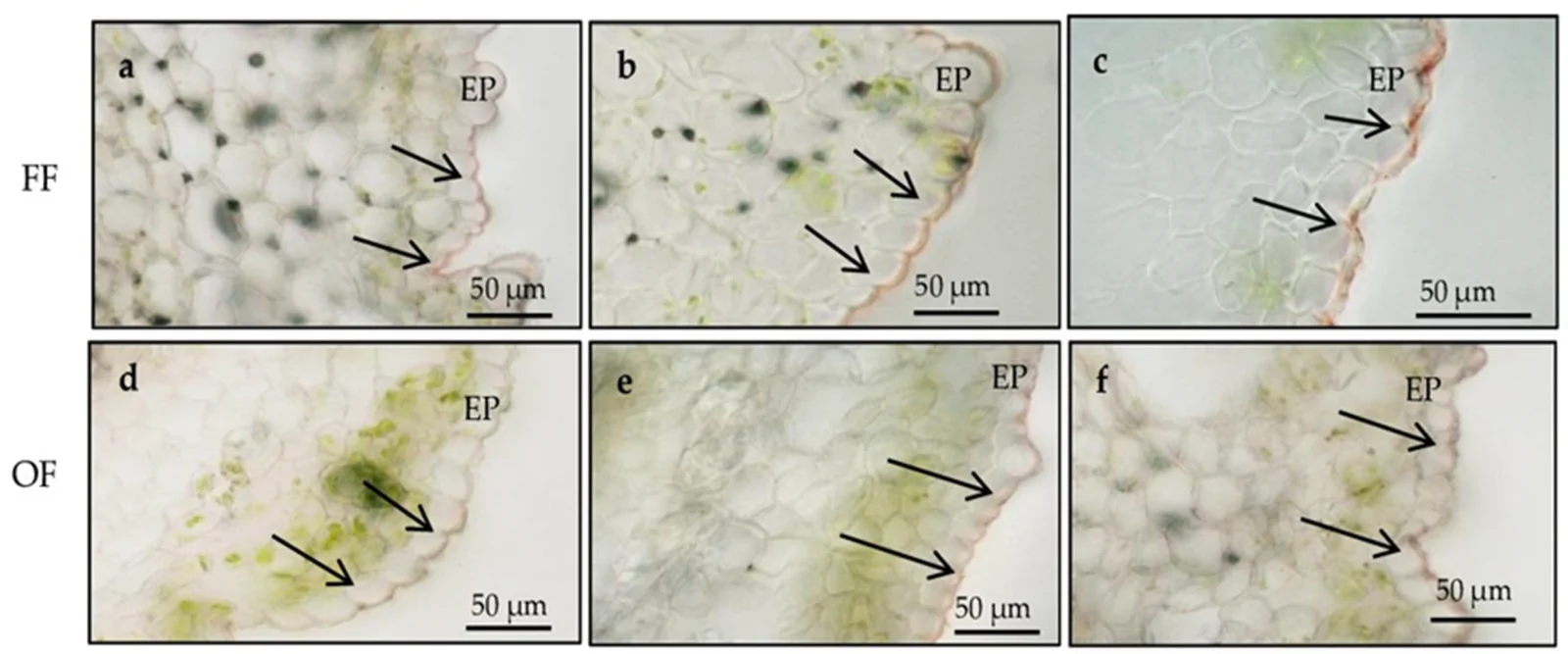
Localization of phenolic compounds (PC) in the stems of in vitro fiber flax (FF) (**a**–**c**) and oilseed flax (OF) (**d**–**f**) seedlings cultivated under control conditions (**a**,**d**) or exposed to 60 μM (**b**,**e**) and 100 μM (**c**,**f**) Cd. Sites of phenolic compound localization are indicated by arrows. Abbreviations: epidermis (EP).

PC are known to play an important role in the defense responses of plant cells owing to their high chemical reactivity, the ability to interact with HMs and mitigate metal-induced toxicity [16]. Therefore, we investigated the compartmentalization of these secondary metabolites in the stems of in vitro seedlings using a histochemical staining procedure with Fast Blue reagent (Figure 12).

In all experimental variants, PC were localized predominantly in the stem EP. Under control conditions, the histochemical staining reaction was more pronounced in FF than in OF. Cultivation of seedlings on Cd-containing media resulted in intensified staining. In FF, this effect was concentration-dependent and was most pronounced at the higher Cd concentration. In OF, staining intensity exceeded the control only slightly at both metal concentrations.

Thus, histochemical analysis of the in vitro-grown seedlings of the two flax varieties revealed similar localization patterns of Cd and PC within the outer stem tissues, while also demonstrating differences between FF and OF in their capacity to accumulate HMs and secondary metabolites.

## 3. Discussion

Cultivated flax (*Linum usitatissimum* L.) has attracted considerable attention not only as a source of valuable plant raw materials and industrial products, but also as a Cd-tolerant species capable of accumulating relatively high concentrations of this metal, including in generative organs and seeds [25,54]. Accumulator plants, which may serve as candidates for phytoremediation, tolerate high HM levels by utilizing such defense mechanisms as intensive production of specific phytochelatins that bind metal ions entering the cytosol, as well as formation of metal-chelate complexes by cytosolic chelating molecules. These include PC, whose biosynthesis—along with other secondary metabolites—reflects the plant’s stress adaptation mechanism during changing environmental conditions [55,56,57].

To date, the vast majority of studies investigating Cd effects in flax have been conducted either on a single variety (FF or OF) or under in vivo conditions, where numerous uncontrolled factors—such as soil composition, rhizosphere microbiota, and climatic variability—substantially complicate the identification of genotype-specific stress responses. In contrast, cultivation under standardized in vitro conditions allows the assessment of genotypic responses of plant tissues independently of environmental influences, while ensuring experimental reproducibility and precise control of pollutant concentrations in the culture medium. In this context, the present study represents the first comprehensive comparative investigation of in vitro-grown seedlings of the two principal flax varieties—FF and OF—integrating analyses of growth characteristics, Cd accumulation, LPO, and PC production. This approach enables us not only to identify variety-specific responses of flax plants to Cd exposure, but also to evaluate the practical potential of each variety for phytoremediation and biotechnological applications.

It is known that, despite the high reproducibility of the results, the control of external conditions and the concentration accuracy in experimental modeling of the in vitro system do not fully replicate all the parameters of the soil system and the properties of the soil-absorbing complex. Indicators such as absorption capacity, water-retention capacity, bioavailability of macro- and micronutrients, and the interaction of the rhizosphere with soil microbiota can significantly modulate plant responses in vivo [5]. Therefore, the results obtained in the in vitro system and their extrapolation to vegetation or field experiments will make it possible to assess the species-specific nature of the response reactions and the adaptive strategies of the two varieties under in situ conditions.

### 3.1. Morphophysiological Characteristics of In Vitro-Grown Flax Seedlings

The in vitro FF and OF seedlings differed in their morphophysiological characteristics, with OF exhibiting greater shoot height and leafiness than FF (Table 1). These differences reflect the intraspecific differentiation of cultivated flax that has arisen through natural and artificial selection and underlies the distinct growth patterns, developmental characteristics, and productivity traits of the two flax varieties [58].

Exposure to Cd resulted in reduced growth parameters of the seedlings, with the effect being more pronounced in OF and at the higher concentration (100 μM), which is confirmed by the data of correlation analysis—a significant negative correlation between Cd and root length (r = −0.82), stem length (r = −0.78), foliage (r = −0.87) and water content (r = −0.86) (Table S1). Growth inhibition under Cd stress is a common response observed in many plant species [59]. This effect is likely attributable to the direct influence of the pollutant on cell division and expansion processes, including reduced mitotic activity and prolongation of the mitotic cycle [60,61]. The more pronounced inhibition of growth processes in OF at a concentration of 100 μM Cd is consistent with its ability to accumulate the pollutant more intensively compared to FF. The high Cd content in OF tissues relative to FF apparently causes disruptions in cellular metabolism, which phenotypically manifests as a decrease in the analyzed growth parameters.

Stem leafiness is an important indicator of plant genotype and intraspecific differentiation [62]. Under control conditions, this parameter was significantly lower in FF than in OF. According to the literature, FF is characterized by a negative correlation between leafiness and fiber content [63]. Exposure to Cd reduced stem leafiness in both varieties, with the effect being more pronounced in OF compared to FF (Table S1). These changes are most likely attributable to variety-specific traits and possible differences in Cd tolerance [32]. These species-specific features are based on fundamentally different adaptive strategies. OF, with its greater biomass and foliage, acts as a more active accumulator of Cd. On the one hand, this ensures a high potential for the absorption of the pollutant; on the other hand, it makes the above-ground organs (in particular, the leaves) the most vulnerable organs to toxic effects, causing a significant reduction in foliage. In contrast, FF demonstrates an “exclusion” strategy, limiting Cd accumulation, which allows it to maintain relative stability of morphometric parameters even at high metal concentrations (100 μM Cd).

Under in vivo conditions, exposure to this pollutant is often manifested by tissue browning and the development of leaf chlorosis, as reported previously [19,64]. However, no such visible symptoms of Cd toxicity were observed at the organ level in the in vitro-grown seedlings of either flax variety.

Thus, the intraspecific differentiation of flax cultivars determines not only the normal morphology of the plants but also shapes their toxicological and ecological response to the action of the pollutant: OF acts as an accumulator, carrying a high risk of damage to tissue structures, whereas FF demonstrates greater resistance due to limited uptake of the toxicant.

### 3.2. Cadmium Content in In Vitro-Grown Flax Seedlings

Flax is recognized as a HM-tolerant species with a pronounced capacity to absorb Cd from soils and nutrient solutions [25,33,65]. Cultivation on substrates containing 4–4000 μM Cd resulted in only limited growth inhibition, reflecting the species’ genetic resistance to Cd stress and its moderate tolerance, with a tolerance index ranging from 40% to 62% [66]. Flax seedlings, in particular, can tolerate Cd concentrations of up to 100 μM with only minor changes in hypocotyl biomass [38]. Even within a single flax variety, the capacity to accumulate HMs, including Cd, varies considerably, as demonstrated by marker-assisted selection studies targeting metal tolerance [65]. The mechanism underlying this resistance is based on maintaining low intracellular concentrations of free Cd through the chelation of most metal ions entering the cell by proteins, low-molecular-weight thiol peptides, phytochelatins, as well as organic acids and PC [19,67]. However, prior to the present study, the extent to which Cd accumulation capacity differs between the two principal flax varieties under controlled in vitro conditions had remained unclear.

The data obtained in the present study demonstrate clear differences in Cd content between the in vitro-grown seedlings of the two flax varieties: Cd levels in OF were nearly 40% higher than in FF at both concentrations tested (Figure 2). OF’s greater metal extraction capacity relative to FF under in vivo conditions has also been reported previously [68,69]. Our study is the first to confirm this pattern in a standardized in vitro system, thereby eliminating the influence of soil and climatic factors.

The higher Cd content observed in the aboveground organs of OF seedlings may be associated with the greater net photosynthetic productivity and dry matter accumulation rate characteristic of this variety [70,71]—traits promoting enhanced uptake and translocation of mineral elements from the nutrient medium, including metal ions. Importantly, the greater Cd accumulation in OF was largely independent of its external concentration within the range examined. This finding suggests that differences in Cd uptake between the two flax varieties are determined primarily by intrinsic physiological and genetic factors and therefore represent a stable varietal trait.

The differences in Cd accumulation between OF and FF reflect the different biochemical strategies they employ to adapt to the effects of the pollutant. The higher Cd accumulation in OF tissues appears to be associated with the intensification of thiol metabolism, the synthesis of phytochelatins and metallothioneins, which ensures effective vacuolar sequestration and reduces the pool of free cytotoxic Cd ions [10,11]. Conversely, a lower Cd content in FF seedlings may indicate the functioning of a “barrier” mechanism at the level of cell walls. For FF, whose metabolism is directed towards fiber formation, it is important to preserve the structural integrity of pectin and cellulose polymers. The limitation of apoplastic Cd transport in FF may be associated with a higher degree of methylesterification of cell wall pectins or with enhanced binding of metal ions by the functional groups of biopolymers, which hinders their transmembrane uptake [33]. Thus, the adaptive OF strategy is based on biochemical detoxification ex planta and in planta, while maintaining a high metabolic status, whereas the FF strategy is aimed at limiting the pathways through which pollutants enter metabolic structures.

### 3.3. Malondialdehyde Content in In Vitro-Grown Flax Seedlings

All plant organisms are characterized by a certain basal level of LPO, commonly assessed by measuring MDA content [72]. Comparative analysis of MDA levels in the in vitro seedlings revealed substantial differences even under control conditions: MDA content in FF was nearly twice that observed in OF (Figure 3). These differences may be attributable to variation in the functional activity of their antioxidant systems, including both enzymatic and non-enzymatic (low-molecular-weight) components of antioxidant defense [73].

Exposure to Cd is frequently accompanied by an increase in MDA content in plant tissues, indicating a stress response [13,74]. However, the presence of Cd in the nutrient medium generally did not result in statistically significant changes in MDA levels in the in vitro flax seedlings relative to the control, which is confirmed by the absence of both positive and negative statistically significant correlations (r < 0.6, at *p* > 0.05) (Table S1). The only exception was OF seedlings cultivated in the presence of 100 μM Cd, which exhibited a significant increase in MDA content, indicative of oxidative stress (Figure 3). This response is most likely related to OF’s greater capacity to accumulate the pollutant. It may be assumed that the intracellular metal burden exceeded the buffering capacity of the antioxidant system at this concentration, resulting in enhanced generation of ROS and, consequently, increased membrane lipid damage and LPO.

All these findings indicate that the development of Cd-induced oxidative stress in the in vitro seedlings is both variety-specific and concentration-dependent. OF, which accumulates greater amounts of the metal, appears to be more susceptible to supra-threshold Cd concentrations than FF, which, despite its higher basal MDA level, exhibited greater stability of this parameter under Cd stress. These results are consistent with previous reports demonstrating the concentration-dependent effects of abiotic stressors, including HMs, on in vitro plant cultures [18,24,34].

The data obtained allow for a deeper understanding of the various biochemical strategies used by the two flax varieties to overcome Cd phytotoxicity. The lower baseline level of MDA in OF under control conditions, combined with its higher capacity for Cd accumulation, indicates that OF relies on an effective but non-renewable (exhaustible) system of intracellular sequestration. Under moderate stress, the incoming Cd^2+^ ions are likely to be effectively chelated and transported into the vacuole, which protects cell membranes from damaging oxidative processes [11,12]. However, at a threshold concentration of 100 μM, this chelation system becomes depleted. An excess of free intracellular Cd promotes the generation of ROS, leading to an observed increase in MDA content. On the contrary, the physiological status of FF indicates a barrier-protection strategy: the functioning of the apoplastic barrier in combination with the restriction of metal intake. The initially high LPO level in FF may be associated with the active signaling function of ROS, which, combined with limiting metal intake into the symplast, prevents an increase in LPO levels even at 100 µm Cd. Thus, if the OF effectively accumulates metal until the chelating systems reach saturation and is susceptible to further stress development, then the FF prioritizes metabolic stability, preventing subsequent stress generation.

### 3.4. Phenolic Compound Accumulation Profile in In Vitro-Grown Flax Seedlings

PC are among the most widespread secondary metabolites, occurring in virtually all plant cells and tissues [16]. Their content and composition are species- and organ-specific and are strongly influenced by growth conditions and stress factors [75]. HMs are among the most aggressive abiotic stressors and, when present at supra-threshold concentrations, act as potent inducers of secondary metabolism, including PC biosynthesis [76,77]. This is largely attributable to the ability of PC to scavenge hydroxyl radicals and function as highly effective antioxidants. It is known that cinnamic and p-coumaric acids, which accumulate in flax seedlings in response to Cd exposure, form complexes with HMs and act as highly reactive chelators involved in metal binding and detoxification [33]. However, clear patterns governing the regulatory effects of Cd on phenolic metabolism in representatives of the genus Linum have not yet been established, making a comparative analysis of PC accumulation in the two flax varieties particularly important.

Determination of TPC, which reflects the overall capacity of plant tissues to produce these metabolites, revealed significant differences between the control variants: TPC in OF seedlings was 30% higher than in FF (Figure 4). This difference likely stems from variation in the composition and stoichiometry of the phenolic metabolome and the expression of genes involved in phenolic biosynthesis between the two flax varieties [78,79].

HMs are known to exert a substantial and concentration-dependent influence on plant secondary metabolism. In most cases, lower concentrations stimulate the accumulation of secondary metabolites, whereas higher concentrations inhibit this process [24,44]. In the in vitro flax seedlings exposed to Cd, TPC changed in a manner dependent on both varietal identity and pollutant concentration. At the lower Cd concentration (60 μM), TPC decreased relative to the control in both varieties. At the higher concentration (100 μM), however, TPC exceeded the control level in FF seedlings and remained comparable to the control in OF seedlings. Enhanced production of secondary metabolites in plants exposed to elevated Cd concentrations has also been reported by other researchers [54,80,81].

The increase in TPC observed in FF seedlings exposed to the higher Cd concentration may reflect the antioxidant and protective functions of PC, including the chelation of Cd^2+^ ions in the apoplast, restriction of metal-ion entry into the symplast, and the interaction of highly reactive phenolic radical groups with ROS-derived alkoxyl radicals [82,83]. In addition, flavonoid–metal complexes exhibit enhanced reactivity toward the superoxide anion radical, thereby facilitating rapid ROS scavenging and reducing LPO [84]. PC can also reduce metal ions through the conversion of internal keto groups into carboxylic acids—a reaction whose efficiency depends on metabolite structure and the number of hydroxyl groups present in the molecule [19]. Molecular genetic studies have shown that Cd modulates transcript levels of genes encoding key enzymes of the phenylpropanoid pathway, including shikimate dehydrogenase, phenylalanine ammonia-lyase, tyrosine ammonia-lyase, cinnamyl alcohol dehydrogenase, and chalcone synthase [46,76,85]. Activation of these enzymes is generally associated with increased PC accumulation, including FL, in a wide range of plant species exposed to HM stress, such as saffron, tomato, apple, grapevine, and flax [81].

In cultivated flax, both the content and composition of PC depend on the plant’s origin, varietal affiliation, and cultivation conditions [30,32]. PP belong to the earliest biosynthetically derived phenolic metabolites [46]. In flax, they are represented by both free and bound forms of ferulic, caffeic, and p-coumaric acids [32,86].

Comparison of PP content in the in vitro-grown seedlings under control conditions revealed substantially higher levels in OF, where these metabolites were approximately twice those observed in FF (Figure 5). This finding further confirms the variety-specific nature of phenolic metabolism, already evident at its earliest stages. Distinct patterns of PP accumulation were also observed in FF and OF seedlings exposed to Cd, representing one of the key findings of the present study. In OF, PP content decreased relative to the control at both Cd concentrations, which is confirmed by a negative statistically significant correlation (r = −0.72) (Table S1). This may indicate either the redirection of these metabolites into alternative biosynthetic or conjugation pathways or their active utilization for Cd chelation and ROS detoxification. In contrast, PP levels in FF remained unchanged at 60 μM Cd and increased slightly at 100 μM Cd, indicating concentration-dependent induction of these metabolites, which is confirmed by a positive statistically significant correlation (r = +0.74) (Table S1). These patterns are consistent with the concept of intraspecific differentiation in flax, whereby the two principal varieties employ distinct physiological and genetic strategies for regulating secondary metabolism under stress conditions. The higher initial level of TPC and PP in the control of OF likely provides this variety with a constitutive defense system. The subsequent decrease in the PP pool under Cd exposure indicates their possible involvement in detoxification processes—chelation of Cd ions in vacuoles or neutralization of Cd-induced ROS, which leads to a reduction in PC content. In contrast, the FF strategy is based on an inducible response: this variety activates de novo PP biosynthesis only when 100 μM Cd is reached. Thus, the OF adaptation strategy may be based on the rapid mobilization of the already existing pool of low-molecular-weight antioxidants in PC, while the FF resistance depends on metabolic restructuring in response to a high Cd concentration of 100 μM.

It should also be noted that the contrasting patterns of PP accumulation observed in FF and OF were consistent with the corresponding changes in LPO only in OF, namely, a decrease at the lower Cd concentration and an increase at the higher concentration relative to the control. The presence of PPs in plant cells does not necessarily result in a reduction in stress indicators, as their quantity may be insufficient to neutralize the ROS generated under these conditions, or they may be diverted toward the synthesis of other specialized metabolites, particularly FL.

FL constitute the most diverse and abundant class of PC in plants, exhibiting a wide range of structures and biological functions [47]. Their content and composition are species-specific, influenced by environmental factors, and closely associated with plant tolerance to various stressors, including HMs [24,31].

According to our data, the in vitro flax seedlings cultivated under control conditions differed markedly in FL content, which was significantly higher in OF compared to FF (Figure 6). A similar pattern was observed for PP accumulation, further confirming a link between the PP and FL branches of phenolic metabolism within each flax variety.

Exposure to Cd resulted in a decline in FL content in both flax varieties, with the effect being more pronounced in OF (Figure 6), and this is also confirmed by the data from correlation analysis—statistically significant correlation for FF (r = −0.97) and for OF (r = −0.80). Moreover, FL levels became nearly identical across all Cd-treated variants, indicating a broadly similar phenolic metabolic response of the two flax varieties to HM exposure.

According to previous studies, OF is characterized by a relatively greater enrichment in FL than PP, whereas FF exhibits the opposite pattern, with PPs predominating over FL [32,79]. A comparable distribution of these phenolic classes was observed in the control variants of the in vitro-grown flax seedlings. However, this pattern was altered by Cd exposure, particularly in OF. The most notable change was a pronounced reduction in FL content accompanied by comparatively smaller changes in PP accumulation relative to the control. Importantly, these responses were not dose-dependent, representing a nontrivial aspect of Cd action in plant cells. We suggest that this phenomenon may reflect homeostatic regulation: once a certain toxicity threshold is reached, the secondary-metabolism system either becomes depleted or switches to alternative adaptive strategies that do not require further modification of the FL pool. This interpretation is consistent with the hypothesis of a concentration threshold above which the biochemical response ceases to be linear [87]. In the context of adaptation to cadmium stress, a sharp decrease in FL content in two varieties may indicate their involvement in Cd detoxification. It is known that FL have a high capacity for intracellular chelation of HM, forming stable and less toxic complexes with them, and also neutralizing the most dangerous forms of ROS [84]. Thus, the observed nonlinear decrease in FL content under Cd exposure reflects their high metabolic “value” as antioxidants and chelators: when the threshold concentration of the metal is reached, the accumulated FL are intensively consumed (bound or oxidized), which makes it impossible to detect their de novo formation and leads to an equalization of their content in the two varieties. The correlation analysis (Table S1) indicates that the two flax varieties employ distinct physiological and genetic strategies for regulating phenolic metabolism under stress conditions. In FF, PP induction was accompanied by suppression of FL accumulation, whereas in OF both classes of PC declined in a coordinated manner.

In FF, a strong positive correlation was observed between Cd content and PP levels (r = +0.74), indicating concentration-dependent induction of these metabolites. A similarly strong positive correlation between TPC and PP content (r = +0.77) suggests that PP make a substantial contribution to the total phenolic pool and, consequently, to FF’s adaptive potential under Cd stress. A strong negative correlation between Cd and FL content (r = −0.97) may reflect inhibition of the flavonoid biosynthetic pathway by the metal. Likewise, the negative correlation between FL and PP (r = −0.70) suggests metabolic reallocation between these branches of phenolic metabolism, possibly indicating a reprogramming or pathway shift within the phenolic metabolic network.

As for OF, a moderate positive correlation was found between PP and FL (r = +0.62), as well as between TPC and PP content (r = +0.71), indicating coordinated regulation of the phenylpropanoid and flavonoid branches of biosynthesis. Cd content showed strong negative correlations with both FL (r = −0.80) and PP (r = −0.72), reflecting an overall suppression of phenolic metabolism following metal uptake. However, since PP and FL contents were themselves significantly correlated in OF, these parameters cannot be considered independent, making it difficult to assess their individual contributions to antioxidant defense.

Taken together, the results reveal an integrated, variety-specific pattern of Cd response in in vitro-grown flax seedlings (Table S1). Despite its greater capacity for metal accumulation, OF exhibited a more pronounced stress response at supra-threshold Cd concentrations, as evidenced by elevated MDA levels and reduced PP content. In contrast, FF accumulated less Cd and appeared to maintain more effective antioxidant protection under elevated metal concentrations, as reflected by an increased TPC and the absence of enhanced stress symptoms (indicated by LPO levels).

### 3.5. Anatomical and Morphological Characteristics of In Vitro-Grown Flax Seedlings

The study of plant cell and tissue morphology and structural organization is deemed one of the most informative approaches for understanding plant responses to abiotic and biotic stress, complementing metabolomic, genomic, and transcriptomic investigations conducted using modern genetic and molecular biological tools [88]. According to numerous authors, plant anatomy and the anatomical adaptation of different organs are particularly important for understanding stress physiology [89,90,91,92,93]. Detailed anatomical studies under HM exposure can substantially expand our knowledge of plant adaptation to changing environmental conditions, especially during the early stages of ontogenesis.

The stem is a key organ in flax ontogenesis and is organized into three principal tissue systems: the epidermal (dermal), vascular (conducting), and mechanical (ground tissue) systems. Investigation of its mesostructure can serve both as a means of assessing flax fiber quality and as an indicator of plant responses to stress factors.

According to our observations, the stems of FF seedlings grown under control conditions exhibited greater ontogenetic maturity than those of OF (Figure 8). This was reflected in a narrower CP layer, which includes chlorenchyma cells and the air cavity formed at the site of stele disintegration. In OF, the CP was broader (8–9 cell layers versus 4–5 in FF) and consisted of cells of differing morphology, indicative of slower ontogenetic development. These differences are likely associated with the developmental characteristics of FF, including shorter transitions between developmental stages and tighter synchronization of vegetative and generative growth [34,58].

Hs exposure is known to induce a wide range of abnormalities in stem tissues, including epidermal ruptures, cellular deformation, and decreases or increases in the size of cortical meristem and collenchyma cells due to disruption of cell division and elongation processes [94]. Other reported effects include trichome swelling, reduced parenchyma cell area, thickening of cell walls, and a decrease in the number of tracheary elements within the stem [95,96,97].

In in vivo-grown flax seedlings, Cd exposure is accompanied by alterations in stem anatomy and structural organization because HMs, including Cd, are transported to aerial organs through the vascular system. These changes are particularly evident in vascular tissues and generally manifest as reductions in stem diameter, CP area, xylem vessel number, vascular tissue density, and the abundance of fibrovascular bundles [88,97].

In the present study, however, no such Cd-induced alterations were detected in the stem mesostructure of the in vitro-grown seedlings of either flax variety, most likely owing to their ontogenetic age and the specific features of their anatomical organization. This may reflect the relatively limited degree of tissue differentiation in in vitro seedlings compared with plants of a comparable age grown under in vivo conditions [96,98].

One important criterion for assessing pollutant effects on plant cells is the evaluation of the relative areas of tissue-forming structures and their distribution within the productive regions of the plant [99]. In flax, particularly FF, whose developmental program is oriented toward fiber formation, the most relevant structures are the EC and EF.

At the stem mesostructural level, morphometric variation was observed in the size and relative proportions of epidermal (EP), endodermal (EC), and elementary fiber (EF) cells in both the control and Cd-treated variants of the two flax varieties. In the control variants, these differences likely reflect the intrinsic biological characteristics of FF and OF, whereas in the Cd-treated variants they may be associated with distinct species-specific patterns of metal tolerance and accumulation.

The similar mean cell areas observed within each tissue type in the control variants of the two in vitro-grown flax varieties, together with the nearly twofold differences between EP and EF cells that were common to both FF and OF, are likely attributable to the generally similar anatomical organization of the stem in the two varieties and to the developmental program of Linum plants, oriented toward fiber formation. During this process, intensive EF differentiation within the fibrovascular bundles occurs, a phenomenon that was more pronounced in FF, as noted above (Figure 9 and Figure 10). These findings are consistent with previous reports demonstrating differences among flax subspecies in the number of bast fibers within fibrovascular bundles, with FF having the highest abundance [30].

Exposure to Cd affected mean cell area only in OF, with reductions observed in EC and EF; these effects were concentration-dependent. This response is most likely related to OF’s greater capacity to accumulate the pollutant compared with FF, such that supra-threshold Cd concentrations resulted in measurable alterations in the analyzed parameters.

The areas of small and large cells proved to be more variable than mean cell area in both flax varieties, particularly in the Cd-treated variants.

Under control conditions, differences between the two varieties across tissue types were observed only at the level of large-cell area and were most pronounced in the EP and EC. The larger area of these cells in FF is likely attributable to varietal characteristics of flax.

Under Cd exposure, FF exhibited a concentration-dependent reduction only in the area of large EC, which is confirmed by the data of correlation analysis—a significant negative correlation (r = −0.91) (Table S1) and EF, whereas neither large nor small EP cells showed significant changes under any treatment (Figure 9). Such responses of the analyzed tissue types may represent one mechanism underlying a stress-avoidance strategy, since epidermal and mechanical tissues constitute barriers to Cd transport and can thereby restrict its movement through the apoplast. Similar increases or decreases in the size of epidermal and mechanical tissue cells under Cd exposure have been reported in other plant species [67]. It is also known that the duration of stress exposure influences fiber formation through its effects on the development of bast bundles composed of EF cells [100].

A different pattern was observed in OF: the significant and concentration-dependent reduction in cell area across all tissue types (Figure 9), particularly among large cell EC and EF, which is confirmed by a negative statistically significant correlation (r = −0.89 and r = −0.87, respectively) (Table S1), may point to a stress-tolerance strategy. The preferential alteration of enlarged cells may be associated with the greater degree of hypocotyl tissue hypertrophy in OF relative to FF. This was manifested by a reduction in hypocotyl length under Cd exposure, physiologically compensated by an increase in diameter resulting from the apparent swelling of most tissues, a phenomenon previously described for other crop species [38].

All of the above findings indicate variety-specific differences between the two types of in vitro flax seedlings, as well as distinct anatomical and morphostructural responses to Cd exposure.

As noted above, compartmentalization represents an important mechanism underlying plant adaptation to HM stress [94,101]. According to our data, Cd was localized predominantly within the EP cell layer of the stems of in vitro seedlings of both flax varieties, and the intensity of its accumulation increased at higher metal concentrations in the culture medium (Figure 11).

It is well established that metal accumulation and metal tolerance are distinct and independently varying traits. Accordingly, plant species can be classified into several categories based on their capacity to accumulate and tolerate metals [102]. Considering the differences observed between the two flax varieties in their morphophysiological characteristics, Cd accumulation and epidermal localization capacity, it can be assumed that FF and OF employ different survival strategies under Cd stress. FF may represent a low-accumulating, highly Cd-tolerant type, characterized by limited metal accumulation in seedlings and a stress-avoidance strategy. In contrast, OF appears to belong to the category of plants exhibiting both high Cd accumulation and high Cd tolerance, as evidenced by its higher tissue metal concentrations and a stress-tolerance strategy. These findings support the concept that the two flax varieties differ in their mechanisms of HM accumulation and tolerance, which also depend on both the nature and concentration of the metal. The involvement of proteins participating in metal accumulation and detoxification processes cannot be excluded (i.e., HM transporter proteins, metal-chelating proteins, and Universal Stress Proteins (USPs), which play important roles in plant stress responses) [103]. Also, the ABC and HMA transporter gene families play an active part in Cd uptake. In flax, these families are represented by nine genes orthologous to those identified in other crop species, including Arabidopsis, rice, and maize [40,104].

The important role of PC, which are among the most effective plant defense metabolites against HM stress, should also be emphasized [16]. These compounds function both as powerful antioxidants and as HM chelators, forming complexes with metal ions—a phenomenon clearly seen in the stems of in vitro flax seedlings (Figure 12). PC were detected predominantly in the epidermal tissues of the stems of both flax varieties under both control and Cd-treatment conditions (Figure 12). Such localization is typical, as the accumulation of phenolics in EP tissues fulfills a protective function, including defense against pathogens, attenuation of UV radiation, and shielding of internal cellular compartments from excessive light exposure [105].

The intensity of the histochemical reaction for PC increased with growing Cd concentration, although the response remained relatively weak because the baseline content of these metabolites in in vitro seedlings was lower than that typically observed under in vivo conditions. The more pronounced PC staining in OF’s EP cells under Cd exposure is likely attributable to its higher endogenous phenolic content compared with FF. The localization of Cd within the EP cell layer of the stem, together with the increased intensity of the PC reaction observed with rising metal concentrations in both flax varieties, is most likely associated with the toxic effects of Cd, which stimulate the production of low-molecular-weight antioxidant metabolites [36,106].

## 4. Materials and Methods

### 4.1. Plant Material and Experimental Conditions

The study was conducted using in vitro seedlings of two flax (*Linum usitatissimum* L.) varieties: FF (cv. Lenok) and OF (cv. Sanlin). Seeds were obtained from the collection of the Federal Research Center for Bast Fiber Crops (Torzhok, Russia). The seeds were surface-sterilized with a 0.1% mercuric chloride solution, for 3 min and then rinsed five times with sterile distilled water, and placed on hormone-free agar-solidified Murashige and Skoog medium [107], containing 0.7% agar (Bacto Agar Typ USA, Ferak, Berlin, Germany) and 2% sucrose (Chimmed, Moscow, Russia). To germinate the seeds, they were sown in groups of 7 in sterile glass test tubes with a volume of 25 mL, containing 10 mL of agar-based nutrient medium.

In the experimental treatments, the basal medium was supplemented with Cd(NO_3_)_2_·7H_2_O (Sigma-Aldrich, St. Louis, MO, USA) at concentrations of 60 or 100 μM, selected on the basis of preliminary experiments. Seedlings were cultivated in sterile glass culture tubes closed with cotton-gauze plugs to ensure passive gas exchange in a growth chamber at 24 °C and a relative air humidity of 70% under a 16 h photoperiod with a photosynthetic photon flux density of 50 μmol·m^−2^·s^−1^ for 21 days. The lighting was provided by fluorescent lamps (OSRAM L 36 W/865, Munich, Germany). Shoot tissues were harvested, immediately frozen in liquid nitrogen, and stored at −70 °C until biochemical analyses were performed.

### 4.2. Determination of Morphometric Parameters of In Vitro Flax Seedlings

Primary root length, shoot height, and leaf number were measured according to standard protocols [108]. Twenty to twenty-five plants were analyzed for each treatment.

### 4.3. Determination of Water Content in In Vitro Flax Seedlings

Tissue water content was determined after drying plant material to constant weight at +70 °C and was calculated using the standard formula [109]:

Water content (%) = [(Fresh weight − Dry weight)/Fresh weight] × 100

### 4.4. Determination of MDA Content in In Vitro Flax Seedlings

MDA content, used as an indicator of LPO level, was determined using the thiobarbituric acid (TBA) (Merck, Darmstadt, Germany)assay [110]. Frozen plant material was homogenized in 0.1 M Tris-HCl buffer (pH 7.5) containing 0.35 M NaCl and 0.5% TBA in 20% aqueous trichloroacetic acid. The homogenate was incubated in a boiling water bath (100 °C, 30 min), centrifuged (16,000× g, 10 min), and the resulting supernatant was used for spectrophotometric analysis (Specord M40, Carl Zeiss, Jena, Germany) at 532 nm [108]. MDA content (μmol·g−1 DW) was calculated using the molar extinction coefficient of 156 mM−1·cm−1 [111].

### 4.5. Determination of Different PC Classes in In Vitro Flax Seedlings

PC were extracted from liquid nitrogen-frozen plant material with 96% ethanol at 45 °C for 45 min (thermostat Gnom, Moscow, Russia) [108]. The extracts were centrifuged (13,500× *g*, 10 min) (centrifuge Minispin, Göttingen, Germany), and the supernatants were used for spectrophotometric analyses (see Section 4.4). TPC was determined using the Folin–Ciocalteu reagent (Panreac, Barcelona, Spain, E.U.) at 725 nm [112]. FL content was measured using a 1% aqueous aluminum chloride solution at 415 nm [113], whereas PP content was determined by direct spectrophotometric measurement (see Section 4.4.) of the extracts at 330 nm [114]. TPC was expressed as mg gallic acid equivalents per gram dry weight (mg GAE·g^−1^ DW), FL content as mg rutin equivalents per gram dry weight (mg RE·g^−1^ DW), and PP content as mg caffeic acid equivalents per gram dry weight (mg CAE·g^−1^ DW).

### 4.6. Determination of Cd Content in In Vitro Flax Seedlings

Plant material was rinsed with distilled water, dried to constant weight at 70 °C, ground, and subjected to acid digestion and thermal mineralization according to standard procedures for plant samples [115]. Cd content was determined using an AA-7000 atomic absorption spectrophotometer (Shimadzu, Kyoto, Japan) and expressed as μg·g^−1^ dry weight. The instrument was calibrated using a series of Cd(NO_3_)_2_ standard solutions prepared from the certified cadmium standard solution GSO 7874-2000 (UNIIM, Yekaterinburg, Russia) [116,117].

### 4.7. Determination of the Localization of PC and Cd in In Vitro Flax Seedlings

For histochemical analyses, stem cross sections (70 μm thick) were prepared using a Thermo Scientific Microm HM650 V vibrating-blade microtome (Thermo Scientific, Dreieich, Germany). PC were visualized using the Fast Blue reagent [118]. Cd localization was assessed using the dithizone staining method [36,53]. Sections were examined with an Axio Imager D1 light microscope (Carl Zeiss, Oberkochen, Germany) and photographed using a Canon A520 digital camera (Tokyo, Japan).

### 4.8. Analysis of Stem Mesostructure in In Vitro Flax Seedlings

To investigate cell morphology and dimensions, stem cross sections (50 μm thick) were prepared using a Microm HM 650 V vibrating-blade microtome (Thermo Scientific, Dreieich, Germany). Section thickness was 50 μm. The areas of 100 cells belonging to the elementary fibers (EFs), epidermis (EP), and endodermis (EC) were analyzed. Observations were conducted under transmitted light using an Axio Imager D1 microscope (Carl Zeiss, Oberkochen, Germany) and Zeiss ZEN software version 10.0 (ZEN Black Edition, Carl Zeiss) [119]. Micrographs were acquired with an AxioCam MRc camera (Carl Zeiss). Morphometric measurements were performed using ZEN lite 2012 software (Carl Zeiss). At least 50 micrographs were analyzed for each of the three biological replicates. Mean areas of both small and large cells were calculated.

### 4.9. Statistical Analysis

All biochemical measurements were performed using three biological replicates and three analytical replicates. Data were statistically processed using Microsoft Excel 2010 14.0 (Redmond, WA, USA) and SigmaPlot 12.2 (Technology Networks, Sudbury, UK). Results are presented in the figures as arithmetic means ± standard deviation (±SDs). Statistical analyses were conducted using two-way analysis of variance (ANOVA). Mean separation was performed following assessment of normality using the Shapiro–Wilk test and subsequent pairwise multiple comparisons using Tukey’s test. The significant differences at *p* < 0.05 are indicated by different Latin letters: uppercase letters denote significant differences between flax varieties, arranged alphabetically from the highest to the lowest value, whereas lowercase letters denote significant differences among Cd treatments, likewise arranged alphabetically from the highest to the lowest value.

## 5. Conclusions

The results presented in this study reveal a coherent, variety-specific pattern of responses to Cd stress in in vitro flax seedlings. Although the oilseed variety (OF) exhibited a greater capacity for Cd accumulation, it also showed a more pronounced stress response at supra-threshold metal concentrations, as evidenced by elevated MDA levels and reduced PP content. In contrast, the fiber variety (FF) accumulated less Cd and appeared to provide more effective antioxidant protection under elevated Cd exposure, as reflected by increased TPC and the absence of enhanced LPO. The differences observed between the two varieties can be interpreted within the framework of contrasting adaptive strategies. OF, characterized by higher productivity and a faster rate of dry matter accumulation, may preferentially adopt a strategy of Cd uptake and compartmentalization. FF, in turn, appears to rely more heavily on phenolic-mediated defense mechanisms, effectively limiting both intracellular Cd accumulation and oxidative stress. These divergent strategies are consistent with the distinct breeding histories of the two flax varieties. FF has been selected primarily for stem fiber quality, a trait that may be associated with enhanced cell wall phenolic metabolism, whereas breeding for seed oil content in OF may have favored metabolic pathways less directly linked to PC biosynthesis. From a practical perspective, OF exhibits greater metal-sorption capacity, whereas FF is characterized by a more pronounced phenolic compound-mediated defense response. Future studies employing transcriptomic and metabolomic approaches will make it possible to identify key candidate genes and metabolic pathways activated in response to cadmium stress in two flax varieties. In particular, transcriptomic analysis will help to identify differentially expressed genes (DEGs) responsible for metal transport (families HMA, NRAMP and ABC), their vacuolar compartmentalization, as well as the regulation of lignin biosynthesis and PP pathways [40]. Parallel metabolomic analysis will make it possible to create a profile of secondary metabolites and identify specific phenolic compounds (including lignans, FL and PP) that are directly involved in Cd chelation and ROS detoxification. The multi-omics approach will open up the possibility of accurately mapping the regulatory networks that determine species-specific resistance strategies and will serve as the basis for marker-assisted selection (MAS) and genetic editing of flax plants with predetermined accumulation properties. These variety-specific traits have important scientific and practical implications for the development of a biotechnological platform based on in vitro-grown flax seedlings as potential HM chelators and sources of phenolic antioxidants. The established species-specific responses of two flax varieties open up prospects for designing a two-component closed-loop biotechnological platform based on in vitro flax cultures. On the one hand, OF seedlings can be used in controlled hydroponic or bioreactor systems as biofilters (chelators). On the other hand, FF seedlings subjected to Cd-induced stress can serve as a renewable source of target secondary metabolites.

## Figures and Tables

**Table 1:** **Morphophysiological parameters of in vitro fiber flax (FF) and oilseed flax (OF) seedlings cultivated under control conditions (Control) or exposed to different concentrations of cadmium (Cd).**

Parameter	FF			OF		
	Control	60 μM Cd	100 μM Cd	Control	60 μM Cd	100 μM Cd
Root length, cm	5.7 ± 0.7 ^Ca^	5.2 ± 0.6 ^Ca^	4.5 ± 0.4 ^CDab^	10.1 ± 1.1 ^Aa^	8.4 ± 0.8 ^Bb^	6.5 ± 0.7 ^Cc^
Shoot length, cm	9.5 ± 0.8 ^Ca^	8.3 ± 0.8 ^Ca^	6.6 ± 0.5 ^Db^	17.5 ± 1.7 ^Aa^	15.2 ± 1.5 ^Aa^	11.1 ± 1.2 ^Bb^
Average number of leaves, pc	7.2 ± 0.8 ^Ba^	6.2 ± 0.7 ^BCa^	5.1 ± 0.6 ^CDab^	10.8 ± 1.2 ^Aa^	8.4 ± 0.8 ^Bb^	7.5 ± 0.8 ^Bb^
Water content, %	92.6 ± 0.19 ^Aa^	91.1 ± 0.78 ^ABb^	90.1 ± 0.65 ^Bb^	93.1 ± 1.27 ^Aa^	90.2 ± 1.13 ^Bb^	89.3 ± 1.1 ^Bb^

Note: Data are presented on a per-seedling basis. Results are expressed as mean ± standard deviation (SD), n = 20. Significant differences at *p* < 0.05 are indicated by different Latin letters: uppercase letters denote significant differences between flax varieties, arranged alphabetically from the highest to the lowest mean value, whereas lowercase letters denote significant differences among cadmium (Cd) treatments, arranged alphabetically from the highest to the lowest mean value. The color shading reflects the normalized values for each parameter: the maximum value across all groups (both varieties, all concentrations) is taken as 100%, the minimum as 38%. Green corresponds to values close to the maximum, yellow to the minimum.

**Table 2:** **Mean cell area (μm^2^) of different stem tissue types in in vitro fiber flax (FF) and oilseed flax (OF) seedlings cultivated under control conditions (Control) or exposed to different concentrations of cadmium (Cd).**

Type of Tissue	FF			OF		
	Control	60 μM Cd	100 μM Cd	Control	60 μM Cd	100 μM Cd
EP	317.9 ± 95.4^Aa^	330.2 ± 79.7^Aa^	328.9 ± 80.3^Aa^	305.3 ± 80.3^Aa^	308.6 ± 90.3^Aa^	289.5 ± 66.7^Aa^
EC	451.5 ± 102.5^Aa^	367.4 ± 83.8^Aa^	404.8 ± 81.7^Aa^	480.9 ± 100.1^Aa^	386.8 ± 80.9^Aa^	348.1 ± 73.3^Aab^
EF	622.6 ± 105.9^Aa^	594.7 ± 95.6^Aa^	693.8 ± 108.4^Aa^	574.1 ± 105.2^Aa^	591.9 ± 109.6^Aa^	374.5 ± 80.6^Bb^

Results are presented as mean ± standard deviation (SD), n = 100. Significant differences at *p* < 0.05 are indicated by different Latin letters: uppercase letters denote significant differences between varieties, arranged alphabetically from the highest to the lowest mean value, whereas lowercase letters denote significant differences among cadmium treatments, arranged alphabetically from the highest to the lowest mean value. Abbreviations: epidermis (EP); endodermal cells (ECs); elementary fibers (EFs).

## Data Availability

The original contributions presented in this study are included in the article/Supplementary Material. Further inquiries can be directed to the corresponding author.

## Supplementary Materials

The following supporting information can be downloaded at: https://www.mdpi.com/article/10.3390/molecules31173064/s1. Table S1: Pearson correlation coefficient matrix between morphoanatomical and physiological-biochemical parameters of different types of in vitro seedlings: fiber flax (FF) (a) and oilseed flax (OF) (b). Cd—cadmium concentration in the medium; FLs—flavonoids; TPC—total phenolic content; PPs—phenylpropanoids; MDA—lipid peroxidation level. Coefficients significant at *p* < 0.05 are highlighted in green.

## Abbreviations

The following abbreviations are used in this manuscript:

PCs	phenolic compounds
TPCs	total phenolic compounds
FF	fiber flax
OF	oilseed flax
HMs	heavy metals
Cd	cadmium
PPs	phenylpropanoid
FLs	flavonoid
LPO	lipid peroxidation
