# Supplementary material for: Morphological Traits, Accumulation and Compartmentalization of Phenolic Compounds and Cadmium (CD2+) in Linum usitatissimum L. Seedlings Under In Vitro Conditions: A Comparative Study of Two Varieties
Source: Molecules. 2026 Aug 31;31(17):3064. doi: 10.3390/molecules31173064 (PMC13567669; doi:10.3390/molecules31173064)
Supplement: Supplementary file 1 [file molecules-31-03064-s001.zip › molecules-4470558-supplementary.pdf]

a Parameter		Cd	Root length	Shoot length	Average number of leaves	Water content	MDA	TPC	PP	FL	Epidermis		Endodermal cells		Elementary fibres	
											Small cells	Large cells	Small cells	Large cells	Small cells	Large cells
Cd		1,00	-0,67	-0,83	-0,79	-0,89	-0,01	0,19	0,74	-0,97	-0,01	-0,06	-0,06	-0,91	0,85	-0,45
Root length			1,00	0,76	0,83	0,77	0,41	-0,06	-0,22	0,37	0,86	0,86	0,59	0,60	0,09	0,84
Shoot length				1,00	0,97	0,89	0,40	-0,19	-0,48	0,61	0,66	0,79	0,51	0,77	-0,17	0,79
Average number of leaves					1,00	0,88	0,42	-0,12	-0,42	0,59	0,71	0,80	0,57	0,78	-0,16	0,83
Water content						1,00	0,38	-0,01	-0,30	0,52	0,77	0,82	0,65	0,76	-0,09	0,89
MDA							1,00	-0,28	-0,10	-0,11	0,46	0,53	0,24	0,09	0,28	0,26
TPC								1,00	0,77	-0,14	0,20	-0,06	0,67	0,13	0,01	0,44
PP									1,00	-0,70	0,24	-0,03	0,44	-0,47	0,57	0,07
FL										1,00	-0,02	0,02	0,04	0,88	-0,85	0,41
Epidermis	Small cells										1,00	0,82	0,95	0,54	0,19	0,94
	Large cells											1,00	-0,38	-0,03	0,15	-0,19
Endodermal cells	Small cells												1,00	0,46	0,02	0,84
	Large cells													1,00	-0,85	0,58
Elementary fibres	Small cells														1,00	-0,10
	Large cells															1,00

b Parameter		Cd	Root length	Shoot length	Average number of leaves	Water content	MDA	TPC	PP	FL	Epidermis		Endodermal cells		Elementary fibres	
											Small cells	Large cells	Small cells	Large cells	Small cells	Large cells
Cd		1,00	-0,82	-0,78	-0,87	-0,86	0,04	-0,48	-0,72	-0,80	-0,04	-0,29	0,14	-0,89	0,23	-0,87
Root length			1,00	0,99	0,97	0,95	-0,22	0,11	0,37	0,62	0,59	0,77	0,44	0,92	0,28	0,96
Shoot length				1,00	0,93	0,91	-0,31	0,03	0,32	0,60	0,65	0,82	0,50	0,87	0,38	0,97
Average number of leaves					1,00	1,00	0,02	0,26	0,47	0,62	0,46	0,63	0,30	0,99	0,06	0,90
Water content						1,00	0,07	0,27	0,48	0,58	0,45	0,62	0,29	0,99	0,04	0,89
MDA							1,00	0,35	0,29	-0,28	-0,35	-0,43	-0,37	0,13	-0,67	-0,32
TPC								1,00	0,71	0,29	-0,50	-0,38	-0,58	0,35	-0,68	0,13
PP									1,00	0,62	-0,36	-0,16	-0,49	0,54	-0,53	0,46
FL										1,00	-0,05	0,15	-0,17	0,61	-0,17	0,67
Epidermis	Small cells										1,00	0,95	0,98	0,35	0,85	0,48
	Large cells											1,00	0,90	0,53	0,81	0,70
Endodermal cells	Small cells												1,00	0,19	0,89	0,32
	Large cells													1,00	-0,07	0,86
Elementary fibres	Small cells														1,00	0,27
	Large cells															1,00

Table S1. Pearson correlation coefficient matrix between morphoanatomical and physiological-biochemical parameters of different types of in vitro seedlings: fiber flax (FF) (a) and oilseed flax (OF) (b). Cd – cadmium concentration in the medium; FL – flavonoids; TPC – total phenolic content; PP – phenylpropanoids; MDA – lipid peroxidation level. Coefficients significant at $p < 0.05$ are highlighted in green.
